# Tunable hydrogel viscoelasticity modulates human neural maturation

**DOI:** 10.1126/sciadv.adh8313

**Published:** 2023-10-20

**Authors:** Julien G. Roth, Michelle S. Huang, Renato S. Navarro, Jason T. Akram, Bauer L. LeSavage, Sarah C. Heilshorn

**Affiliations:** ^1^ Institute for Stem Cell Biology and Regenerative Medicine, Stanford University School of Medicine, Stanford, CA, USA.; ^2^ Complex in Vitro Systems, Safety Assessment, Genentech Inc., South San Francisco, CA, USA.; ^3^ Department of Chemical Engineering, Stanford University, Stanford, CA, USA.; ^4^ Department of Materials Science and Engineering, Stanford University, Stanford, CA, USA.; ^5^ Department of Bioengineering, Stanford University, Stanford, CA, USA.

## Abstract

Human-induced pluripotent stem cells (hiPSCs) have emerged as a promising in vitro model system for studying neurodevelopment. However, current models remain limited in their ability to incorporate tunable biomechanical signaling cues imparted by the extracellular matrix (ECM). The native brain ECM is viscoelastic and stress-relaxing, exhibiting a time-dependent response to an applied force. To recapitulate the remodelability of the neural ECM, we developed a family of protein-engineered hydrogels that exhibit tunable stress relaxation rates. hiPSC-derived neural progenitor cells (NPCs) encapsulated within these gels underwent relaxation rate-dependent maturation. Specifically, NPCs within hydrogels with faster stress relaxation rates extended longer, more complex neuritic projections, exhibited decreased metabolic activity, and expressed higher levels of genes associated with neural maturation. By inhibiting actin polymerization, we observed decreased neuritic projections and a concomitant decrease in neural maturation gene expression. Together, these results suggest that microenvironmental viscoelasticity is sufficient to bias human NPC maturation.

## Teaser

Human neural progenitor cells exhibit distinct maturation phenotypes as a result of the viscoelasticity of their microenvironment.

## INTRODUCTION

Human brain development is characterized by the differentiation, migration, and integration of cells within a spatiotemporally dynamic microenvironment rich in biochemical and biophysical signaling cues (*1*). These cues are imparted both by cells and the neural extracellular matrix (ECM), composed primarily of hyaluronan (HA), chondroitin sulfate proteoglycans, link proteins, and basement membrane proteins (*2*, *3*). The composition and structure of the ECM influence its mechanical properties, which, in turn, affect a range of developmental processes within the brain (*4*).

Investigations into the effects of biophysical cues on neural cells have relied on two- and three-dimensional (3D) in vitro models with engineered mechanical properties. While matrix stiffness has repeatedly been shown to drive substantial differences in murine neurogenesis (*5*–*9*), the majority of these studies use linearly elastic materials. However, native brain tissue is not a purely elastic material; instead, the brain is viscoelastic and exhibits a time-dependent response to an applied force (*10*, *11*). Viscoelastic materials, such as brain tissue, will undergo stress relaxation, such that when a force is applied, the material can be remodeled to dissipate that force (*12*). While previous studies have demonstrated that stress relaxation affects mesenchymal stromal cell differentiation (*13*), endothelial cell vasculogenesis (*14*), and induced pluripotent stem morphogenesis (*15*), it has been understudied in human models of brain development. Notably, stress relaxation was shown to be a greater driver of transcriptional differences, compared to matrix stiffness and adhesive ligand concentration, in human stem cell-derived neural progenitor cells (NPCs) (*16*). However, to date, no study has characterized the resultant cellular phenotypes regulated by stress relaxation in human NPCs. Given the spatiotemporal variation in brain viscoelasticity (*17*, *18*), it follows that stress relaxation may influence NPC behavior throughout differentiation and cell fate acquisition.

Human-induced pluripotent stem cell (hiPSC)–derived neural cells recapitulate facets of human brain development and show promise as in vitro models of neurodevelopmental and neurodegenerative disorders (*19*, *20*). While human stem cells have begun to be introduced into studies evaluating the effects of biophysical cues on neural development (*16*, *21*, *22*), the majority of such studies have used murine cell lines. Given the anatomical and physiological differences between murine and human brain development (*23*–*25*), as well as the observation that stress relaxation is implicated in human brain development and disease (*17*, *26*), there exists a need for studies to characterize how stress relaxation affects human neural cells over time.

Here, we probe the responses of hiPSC-derived NPCs (hNPCs) encapsulated within viscoelastic microenvironments. While natural biomaterials such as collagen and fibrin exhibit stress relaxation, decoupling their relaxation rate from other biophysical and biochemical cues, namely, stiffness and adhesive ligand density, has proven challenging (*27*). To address this challenge, we develop a family of protein-engineered biomaterials with stress relaxation rates that are tunable independently of their stiffness and adhesive ligand density. We culture human NPCs in 3D within our engineered matrices and characterize the morphological, physiological, and transcriptomic differences that emerge as a function of time and stress relaxation rate. Our results suggest that cell-mediated strain within remodelable fast stress-relaxing gels drives neural maturation and that tuning biomechanical signaling cues within engineered microenvironments has great potential to advance current models of human neurodevelopment.

## RESULTS

### Viscoelastic hydrogels promote neurite extension

To investigate the mechanosensitivity of hNPCs to differences in stress relaxation rate, we designed a series of tunable, viscoelastic 3D matrices consisting of HA and elastin-like protein (ELP), which we term HELP (*28*). HA was chosen as it both is the predominant component of the neural ECM and contributes biochemical signaling cues (*2*). ELP is a recombinant protein composed of alternating elastin-derived sequences and cell-adhesive sequences (*29*). Elastin provides structural support throughout the matrix. For the cell-adhesive sequence, we chose a fibronectin-derived, integrin-binding, arginine-glycine-aspartic acid (RGD) motif recognized by hNPCs (*30*, *31*). The modular, customizable design of ELPs enables the independent control of biochemical and biophysical matrix cues within a 3D hydrogel (*29*, *32*).

**Fig. 1.**
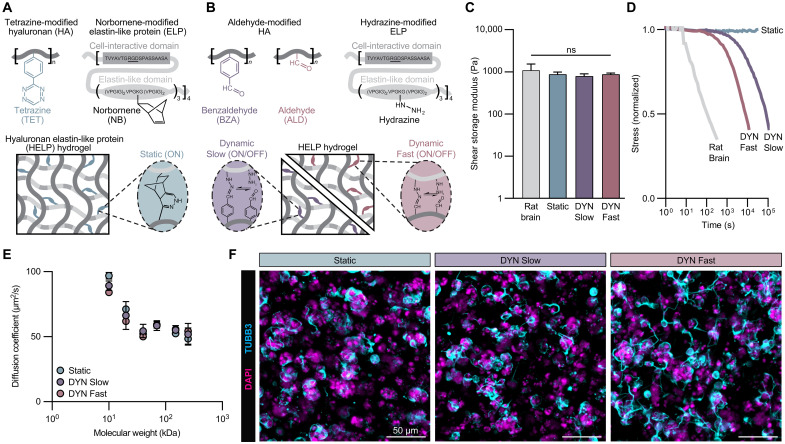
Tunable viscoelastic hydrogels promote hNPC culture and neurite extension. (**A**) Schematic of tetrazine-modified hyaluronan (HA) and norbornene-modified ELP creating a static covalent hydrogel. (**B**) Schematic of aldehyde-modified (either benzaldehyde or aldehyde) HA and hydrazine-modified ELP creating dynamic covalent hydrogels. Dynamic slow (DYN Slow) hydrogels contain only benzaldehyde-modified HA, while dynamic fast (DYN Fast) hydrogels consist of a 1:1 mixture of benzaldehyde- and aldehyde-modified HA. (**C**) Shear modulus of rat brain and all three gel formulations (*N* = 3 to 4). (**D**) Normalized representative stress relaxation curves for rat brain and all three gel formulations. (**E**) Diffusivity of molecules of varying molecular weight through Static, DYN Slow, and DYN Fast hydrogels as measured by fluorescence recovery after photobleaching (FRAP) (*N* = 4). (**F**) Representative maximum projection fluorescence images of hNPCs encapsulated at 3 × 10^4^ cells/μl within all three gel formulations after 7 days of culture with labeled cell nuclei (DAPI, magenta) and neuritic extensions (TUBB3, cyan). Statistical analyses performed as one-way analysis of variance (ANOVA) with Tukey’s multiple comparisons test. Data plotted as mean ± SD. ns, not significant.

To obtain hydrogels with varying rates of stress relaxation, we leveraged both static covalent cross-linking and dynamic covalent cross-linking. Static, elastic HELP gels are fabricated by cross-linking tetrazine-modified HA and norbornene-modified ELP (Fig. 1A and fig. S1, A and B). To form dynamic, stress-relaxing HELP gels, ELP is modified with a hydrazine moiety, and HA is modified with either an aliphatic aldehyde or benzaldehyde group (Fig. 1B and figs. S2, A and B, and S3, A to C). The kinetics of the dynamic covalent cross-links formed upon mixing these two reactive groups are known to depend on the precise chemical structure of the reactive groups, with aldehyde-formed bonds having fast ON/OFF kinetics and benzaldehyde-formed bonds having slow ON/OFF kinetics (*33*, *34*). We engineered these hydrogels to exhibit a similar stiffness of ~800 Pa, which falls within the reported range of shear elastic modulus for native brain tissue and within a range known to support neuronal differentiation (Fig. 1C and fig. S4) (*35*). An overall gelation time of 30 min was sufficient to reach a plateau modulus for all conditions (fig. S4A). Notably, the elastic modulus chosen for these hydrogels is markedly higher than that of the commonly used, commercially available basement membrane matrix derived from decellularized Engelbreth-Holm-Swarm mouse sarcoma (i.e., Matrigel, ~100 Pa) (fig. S4C). We ensure that a constant polymer concentration and cell-adhesive ligand density are maintained across hydrogel conditions by forming all HELP gels with 1 wt % ELP and 1 wt % HA, resulting in a final concentration of 1 mM RGD for each hydrogel.

Independently of elastic modulus and adhesive ligand density, we modulated the stress relaxation rate of our dynamic HELP gels by tuning the composition of aldehyde- and benzaldehyde-functionalized HA within the hydrogel (100% benzaldehyde, “DYN Slow”; 50% aldehyde and 50% benzaldehyde, “DYN Fast”). Because of the differing kinetics of the dynamic bonds, a range of stress relaxation rates were achieved. In response to a constant applied shear strain, DYN Fast gels dissipate stress more quickly compared to DYN Slow gels, whereas Static gels do not relax in response to the applied strain after 24 hours (Fig. 1D). While the stress relaxation rates for these HELP gels remain slower compared to rat brain tissue and Matrigel (Fig. 1D and fig. S4C), this family of HELP gels offers a reproducible and tunable system with respect to biophysical and biochemical cues and thus is able to facilitate the interrogation of how hNPC behavior is affected by matrix viscoelasticity.

HELP gels exhibit several important properties that render them compatible for 3D hNPC culture. A rapid gelation time (<30 min) for all hydrogel conditions limits cell settling, which ensures that the cells are truly experiencing a 3D environment (fig. S4A) (*36*). In addition, previous work has demonstrated that neural cells can tolerate a 30-min encapsulation protocol before receiving nutrient-rich medium (*31*). Another important consideration for 3D hydrogels is the transport of growth factors, neurotrophic factors, and small molecules required for culture viability. Fluorescence recovery after photobleaching (FRAP) studies show that all HELP hydrogel formulations have similar diffusion rates of macromolecules ranging in size from 10 to 250 kDa, ensuring no differences in nutrient or oxygen delivery to hNPCs cultured in the different matrices (Fig. 1E).

We next investigated how hNPC behavior was influenced by matrix stress relaxation rate. hNPCs were encapsulated within HELP gels with varying stress relaxation rates as single cells and cultured in neural medium without any additional mitogens or differentiation factors. After 7 days of culture within the hydrogels, hNPCs were fixed and stained for the neuronal lineage marker βIII-tubulin (TUBB3). Notably, we observed distinct morphological differences depending on the matrix stress relaxation rate, with hNPCs exhibiting varying degrees of TUBB3^+^ neurite extension throughout the gel (Fig. 1F). Specifically, faster stress relaxation rates promoted increased neurite extension, whereas hNPCs in the DYN Slow and Static conditions maintained more rounded cellular morphologies and clustering with limited neurite extension. Given the link between cell morphology and function (*37*–*39*), these results suggested that matrix stress relaxation rate may regulate hNPC behavior and maturation.

### Fast stress relaxation rates drive more mature hNPC physiology and morphology

**Fig. 2.**
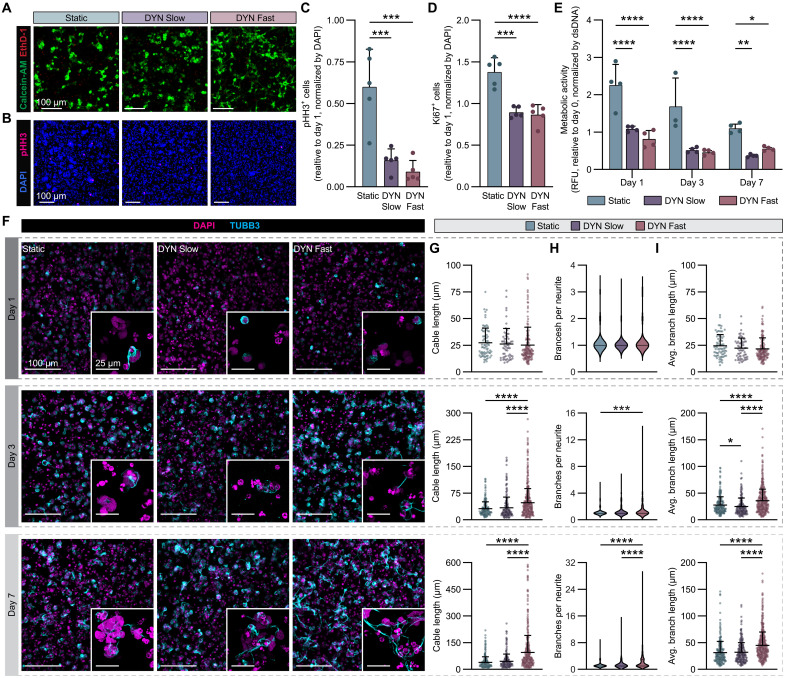
Stress relaxation drives more mature hNPC physiology and morphology. (**A**) Representative maximum projection fluorescence images of hNPCs encapsulated at 3 × 10^4^ cells/μl within all three gel formulations after 7 days of culture with calcein-AM–labeled live cells (green) and ethidium homodimer-1 (EthD-1)–labeled dead cells (red). (**B**) Representative maximum projection fluorescence images of hNPCs encapsulated within all three gel formulations after 7 days of culture with labeled cells currently undergoing M phase of mitosis (pHH3, magenta). (**C** and **D**) Quantification of the number of cells with pHH3 expression (C) and Ki67 expression (D) after 7 days of culture in all three gel formulations relative to 1 day post-encapsulation (*N* = 5 replicate hydrogels). (**E**) Quantification of the relative metabolic activity of encapsulated cells over time. Metabolic activity was normalized by the amount of dsDNA and taken relative to the normalized activity 1 hour post-encapsulation (*N* = 4 replicate hydrogels). RFU, relative fluorescence units. (**F**) Representative maximum projection fluorescence images of hNPC neuritic extension (DAPI-labeled nuclei in magenta, TUBB3-labeled neurites in cyan) over time across all three gel formulations. (**G**) Quantification of neurite cable length over time. Each dot represents a single cell with traceable neurites. (**H**) Quantification of the number of branches per neurite over time. Median and quartiles denoted with a black line. (**I**) Quantification of the average branch length of neurites over time. Each dot represents a single cell with traceable neurites. See table S2 for statistical details for (F to I). Statistical analyses performed as one-way ANOVA with Tukey’s multiple comparisons test (C and D), two-way ANOVA with Dunnett’s multiple comparisons test (E), and Kruskal-Wallis test with Dunn’s multiple comparisons test (G to I). Data plotted as mean ± SD where *P < 0.05, **P < 0.01, ***P < 0.001, and ****P < 0.0001.

To begin to address the possibility that the viscosity of the local microenvironment influences hNPC maturation, we explored the effect of matrix stress relaxation rate on hNPC viability, proliferation, and metabolic activity. Live/Dead staining was performed on days 1, 3, and 7 to assess both acute and long-term cell viability (Fig. 2A and fig. S5A). High viability was observed across all hydrogel conditions at day 1, confirming that the cell encapsulation process is cytocompatible. hNPCs maintained high viability at day 7 despite their inability to extend neurites in the Static and DYN Slow conditions. However, at day 28 post-encapsulation, the population of viable cells within Static gels had substantially decreased, while cells within DYN Slow gels were clustered in large cell aggregates and cells within DYN Fast gels extended particularly long neuritic projections (fig. S5, B and C).

Differences in double-stranded DNA (dsDNA) content and proliferation began to emerge by day 7. Encapsulated hNPCs undergo continuous proliferation in all HELP conditions, as characterized by dsDNA quantification (fig. S6, A and B). However, immunostaining for proliferation markers pHH3 and Ki67 at day 7 revealed that hNPCs in Static gels exhibit significantly higher percentages of cells that remain in an active proliferative state (Fig. 2, B to D, and fig. S6C). As NPCs mature, they transition from primarily self-renewing symmetric cell divisions to terminal asymmetric divisions (*40*, *41*). Neurons are postmitotic and cannot further proliferate; the shift from NPC to neuron is associated with the down-regulation of cell cycle markers (*42*). Consistent with these proliferation data and the known shift in cell cycle dynamics, metabolic activity on a per-cell level was also significantly higher in Static gels compared to DYN Slow and DYN Fast gels (Fig. 2E). Collectively, these data demonstrate that while all hydrogel conditions support hNPC viability, the static, nonstress-relaxing condition increases hNPC proliferation, implying that the non-stress-relaxing matrix may promote self-renewal and the retention of a more progenitor-like state. Meanwhile, the decreased relative number of proliferative cells and metabolic activity observed in the dynamic HELP gels point toward a more mature, postmitotic phenotype.

Next, we sought to study the impact of matrix stress relaxation rate on hNPC morphological maturity over time. To quantify neurite extension, hNPCs were stained for TUBB3, a marker localized to neuritic projections, and imaged on days 1, 3, and 7 (Fig. 2F and fig. S7A). In addition to stress relaxation rate, initial cell density also affects neurite morphology. In DYN Fast gels, hNPCs seeded at a low initial cell density (1 × 10^4^ cells/μl) exhibited low levels of TUBB3 expression and neurite extension by day 7 (fig. S7, A and B). However, at sufficiently high initial cell densities (3 × 10^4^ or 5 × 10^4^ cells/μl), similar degrees of increased TUBB3 expression and neurite extension are observed (fig. S7, A and B). Previous work by our lab identified cell-cell contact, mediated through local matrix degradation by a cell-secreted protease, as an important factor governing murine NPC differentiation capacity (*32*, *36*). It follows that a certain threshold of cell density may be required for human NPCs to undergo morphological changes throughout a 3D matrix. As a result, the remainder of the studies all use an initial cell density of 3 × 10^4^ cells/μl.

Three different metrics were extracted from neurite tracing to quantify neurite length and complexity: (i) cable length, which reflects the total sum of the lengths of all neurites (stemming from the root of the soma to the end of the branches) (Fig. 2G); (ii) branches per neurite, which represents how many intersection points exist for each root (Fig. 2H); and (iii) average branch length, which reflects the average length of all neurites extending from the root (Fig. 2I). On day 1 following encapsulation, hNPCs in all conditions exhibit relatively limited TUBB3 expression, suggesting that the hNPCs have not yet sufficiently remodeled their surrounding matrix (Fig. 2F). There are also no statistically significant differences in any of the three metrics when comparing the three hydrogel conditions at day 1 (Fig. 2, G to I). By day 3, hNPCs express TUBB3 at higher levels, and morphological differences begin to emerge. Specifically, increased TUBB3 expression, cable length, branches per neurite, and average branch length are all observed with faster stress relaxation rates (Fig. 2, F to I). By day 7, these differences are further pronounced, with hNPCs in DYN Fast gels exhibiting significantly longer and more complex neurites, whereas hNPCs in Static gels are unable to extend neurites (Fig. 2, F to I). Together, these data demonstrate that material properties are sufficient to drive changes in neurite length and complexity, with faster stress relaxation rates promoting increased neurite extension.

The remodelability of a cell’s local microenvironment is influenced by both cell intrinsic and extrinsic factors. Given the aforementioned role of cell-mediated matrix degradation in murine NPC differentiation (*32*, *36*), we characterized the relative prevalence of cell-secreted enzymes that could degrade HELP matrices. While hNPCs in all three gel formulations secreted hyaluronidase, no significant differences in hyaluronidase activity were observed (fig. S8A). Moreover, there were no significant differences in the activity of several proteases as a function of gel condition (fig. S8B). Together, the emergent differences in hNPC physiology and morphology were not influenced by cell-mediated matrix degradation.

### Transcriptional differences in NPCs across time and hydrogel remodelability

These material-driven phenotypic differences in hNPC proliferation, metabolism, and morphology drove us to explore potential transcriptional differences. To characterize the transcriptional profile of the encapsulated hNPCs, we performed RNA sequencing (RNA-seq) on the cells in all three gel formulations over two time points. We collected mRNA 1-hour post-encapsulation to serve as a baseline as previous studies have demonstrated that transcriptomic changes in adult rat hippocampal neural stem cells can emerge after 12 hours (*9*). We also collected mRNA at 1-week post-encapsulation to match the latest time point for the phenotypic data. All 18 samples exhibited high replicate-to-replicate correlation (fig. S9).

**Fig. 3.**
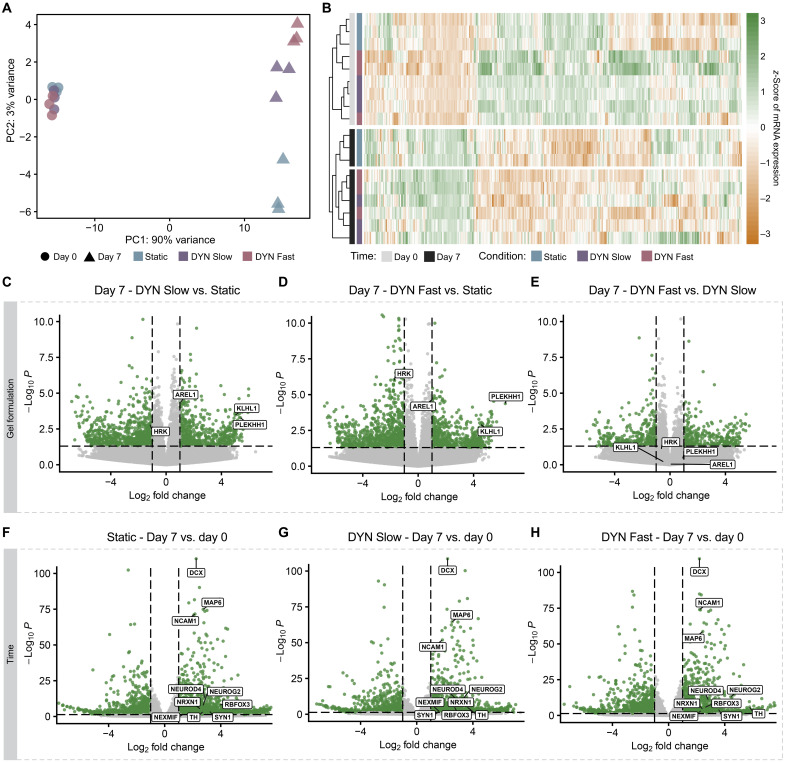
Transcriptional differences emerge in hNPCs across time and hydrogel relaxation rate. (**A**) Principal components analysis of normalized count data reveals that hNPCs encapsulated at 3 × 10^4^ cells/μl within hydrogels cluster first by time and second, after 7 days of culture, by gel formulation (*N* = 3 replicate hydrogels). Axes represent the first two principal components (PC1 and PC2). (**B**) Heatmap of gene expression represented as *z*-scores for the top 500 differentially expressed genes as identified with DESeq2. Counts were normalized and scaled across columns. (**C** to **H**) Volcano plots showing *P* value and fold change for hNPCs encapsulated within the stated hydrogel condition and time point. Differentially expressed genes (fold change ≥ 1, adjusted *P* < 0.05 with false discovery rate) are depicted in green. (C to E) Pairwise comparisons between all three gel conditions at day 7 converge on genes associated with apoptosis (*HRK* and *AREL1*) and cytoskeletal dynamics (*KLHL1* and *PLEKHH1*). (F to H) Pairwise comparisons across the day 0 and day 7 time points for each gel condition. Genes associated with neurogenesis are labeled.

As hNPCs are known to differentiate in 2D over time when cultured in medium devoid of mitogens (*43*), we hypothesized that the gene expression profiles of the encapsulated hNPCs would vary across encapsulation times. The greatest driver of clustering in principle components analysis (PCA) was sample collection time (Fig. 3A). At day 0, all nine samples, regardless of gel condition, were tightly clustered away from the day 7 samples. Notably, the second largest contributor to sample clustering appeared to be gel condition, as the day 7 samples segregated such that the three Static samples were isolated from the six DYN samples. The three DYN Slow samples clustered slightly closer to the Static samples, implying that the faster stress relaxation kinetics (i.e., DYN Fast) resulted in the greatest transcriptomic differences. These results were reinforced by plotting the 500 most differentially expressed genes and arranging the samples with agnostic hierarchical clustering (Fig. 3B). The 100 most differentially expressed genes related to neuronal differentiation and glial differentiation also resulted in the same clustering, with the static samples at day 7 separated from the DYN samples (fig. S10, A and B). Together, the PCA and differential expression analysis suggested that differentiation time and stress relaxation rate drove marked transcriptional variance. Moreover, the material-driven differences in gene expression were most substantial at day 7.

To explore the specific genes that contributed to these differences, we performed differential expression analysis across pairwise comparisons and plotted gene expression by fold change and *P* value. When comparing expression profiles across gel conditions at day 7, the resultant volcano plots revealed two distinct patterns: (i) That the number of differentially expressed genes is higher when comparing the Static to either DYN gel formulation and (ii) that the biological processes most affected at day 7 are apoptosis and cytoskeletal dynamics (Fig. 3, C to E). First, the total number of differentially expressed genes, herein taken to be genes with an adjusted *P* value less than 0.05, was over 500 when the day 7 comparison was made between either DYN gel formulation and the Static formulation (DYN Slow versus Static: 521, DYN Fast versus Static: 556). In comparison, only 101 differentially expressed genes were identified when comparing day 7 between DYN Fast and DYN Slow. Second, when comparing the two DYN gel formulations to the Static formulation at day 7, genes that are associated with increased degrees of apoptosis and cell stress (*HRK*) were up-regulated in Static gels, while genes known to limit apoptosis (*AREL1*) were up-regulated in DYN gels. These differences may underlie the decline in cell viability which emerges over long-term culture within Static HELP gels (fig. S5, B and C). Moreover, genes that impinge upon the neural actin-myosin complex (*PLEHH1* and *KLHK1*) were up-regulated in DYN gels compared to Static gels. These data suggest that the capacity for the gel to undergo stress relaxation may be driving differences in the cytoskeletal dynamics of the encapsulated hNPCs over time.

Across time, the most notable and reproducible differential expression was attributed to genes associated with neurogenesis, neurite extension, and neural maturation (Fig. 3, F to H). For all three gel formulations, the gene that encodes the microtubule-associated protein doublecortin, *DCX*, exhibited notably small *P* values. Doublecortin, a 40-kDa phosphoprotein exclusively expressed in the nervous system, has long been associated with neural migration (*44*), stabilization of neuritic branches (*45*), and regulation of actin in axonal growth cones (*46*). While *DCX* is conventionally associated with NPCs, gene markers of more mature neurons, including *RBFOX3*, *TH*, and *SYN1*, were also significantly increased in cells collected from all three gels at day 7 compared to day 0. This repeated convergence on neural maturation and cytoskeletal regulation within the transcriptome corroborates the observed morphological differences in the encapsulated hNPCs as a function of time. The maturation of NPCs into neurons is also characterized by a known metabolic shift from glycolysis to oxidative phosphorylation (*42*, *47*). To explore this maturation-associated transition in metabolic state, we plotted the 100 most differentially expressed genes associated with the Gene Ontology term “positive regulation of metabolic process” and arranged the samples with agnostic hierarchical clustering (fig. S11A). The samples were primarily clustered by time point, and among the day 7 samples, the Static samples clustered separately from the DYN Fast and DYN Slow samples, indicating a difference in metabolism-related gene expression as a function of gel remodelability. Furthermore, several glycolysis-related genes were up-regulated in the Static samples, whereas many oxidative phosphorylation-related genes were up-regulated in the DYN Fast and DYN Slow samples (fig. S11B). Cell cycle–related genes were also up-regulated in the Static samples (fig. S11B), which supports the observation that NPCs encapsulated within the Static gels remain in a more proliferative, progenitor-like state after 7 days (Fig. 2, B to D, and fig. S6C). Together, these transcriptomic and phenotypic data suggest that the encapsulated hNPCs are differentiating into more mature neurons at different rates depending on the stress relaxation profile of their microenvironment.

### Viscoelastic hydrogels modulate the cell fate acquisition of hNPCs

**Fig. 4.**
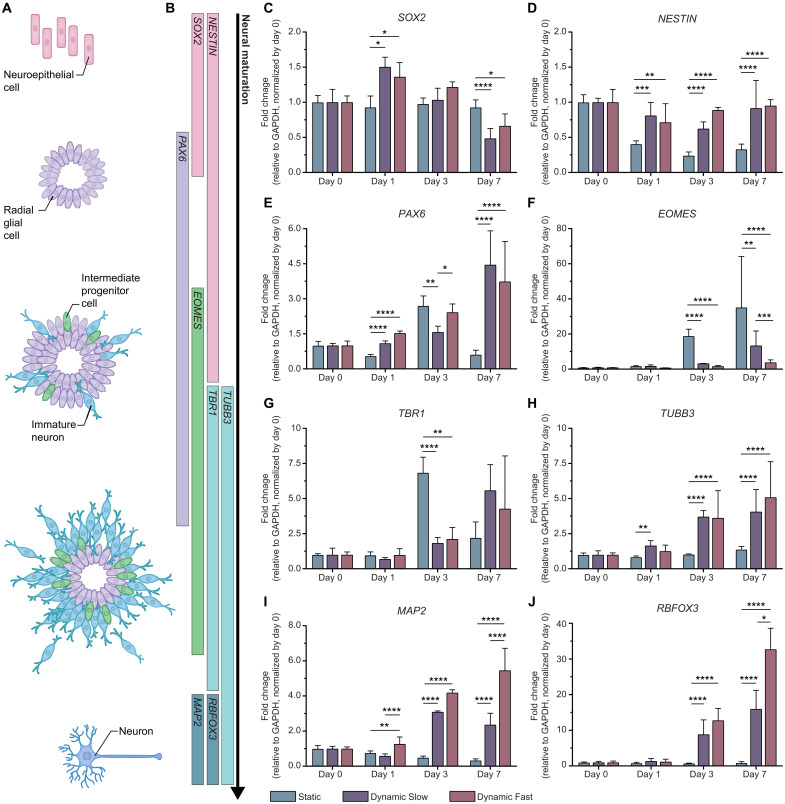
Viscoelastic hydrogels modulate the neuronal maturation of hNPCs. (**A**) Schematic of neural differentiation with color-coded cell fates. (**B**) Canonical gene markers frequently used to identify the cell fates depicted in the schematic to the left. (**C** to **J**) mRNA expression of canonical neural differentiation markers for hNPCs encapsulated at 3 × 10^4^ cells/μl in all three gel formulations over 7 days in culture (*N* = 4 replicate hydrogels). Statistical analyses performed as two-way ANOVA with Šídák’s multiple comparisons test. Data plotted as mean ± SD where *P < 0.05, **P < 0.01, ***P < 0.001, and ****P < 0.0001. GAPDH, glyceraldehyde phosphate dehydrogenase.

To further characterize changes in hNPC cell fate over additional time points within our three gel formulations, we turned to targeted transcriptomic characterization of neural maturation genes. Broadly, hNPCs are known to progress through a series of cell fates from neuroepithelial cells to radial glia to intermediate progenitors to immature neurons and, lastly, to mature neurons (Fig. 4A) (*48*, *49*). These transitions in fate are accompanied by temporal changes in the expression of canonical neural lineage genes (Fig. 4B). Here, we quantify the relative abundance of eight such genes across five stages of neural differentiation at days 0, 1, 3, and 7 post-encapsulation within 3D hydrogels with tunable relaxation rates.

At earlier developmental time points, SRY (sex-determining region)–box 2 (*SOX2*), neuroepithelial stem cell protein (*NESTIN*), and paired box 6 (*PAX6*) are often used as gene markers of NPCs, although their temporal profiles have distinct differences (Fig. 4B). SOX2 is a transcription factor that is expressed throughout the neuroepithelium and regulates the proclivity for NPCs to self-renew or differentiate (*50*, *51*). While *SOX2* expression is consistent over time within the Static gel, by day 7, its expression levels significantly decreased within the two DYN gels (Fig. 4C). NESTIN is a type VI intermediate filament protein that is transiently expressed in NPCs and immature neurons before being replaced by tissue-specific intermediate filament proteins (*52*). *NESTIN* expression is decreased in Static gels across all three time points (Fig. 4D). Given the recent evidence that NESTIN can influence immature neuron axon guidance (*53*), this decrease suggests that differentiating hNPCs within Static gels may be less capable of adopting complex neuritic networks. PAX6 is a transcription factor detected in radial glial cells of the developing forebrain that regulates cell cycle and the acquisition of more mature neural cell fates (*54*, *55*). By day 7, the expression of *PAX6* is significantly higher in the two DYN gels compared to the Static gel (Fig. 4E). These data suggest that a greater proportion of cells within the DYN gel formulations are radial glia. Another possibility is that the relative number of PAX6-expressing cells is similar across gels, but the degree of *PAX6* expression is higher in DYN formulations. Recent studies in which *PAX6* was overexpressed in murine embryonic stem cells and adult hippocampal progenitor cells suggest that increased *PAX6* expression may drive both accelerated neural differentiation and increased neuronal migration, respectively (*56*, *57*).

*PAX6*-expressing radial glial cells give rise to immature neurons either directly through neurogenesis or indirectly through the transient formation of transit-amplifying intermediate progenitor cells (*58*). These intermediate progenitor cells can be distinguished from radial glia by their expression of T-box brain protein 2 (TBR2), which is encoded by the gene eomesodermin (*EOMES*). Notably, the expression of *EOMES* is significantly increased over 20-fold in hNPCs within Static gels at both day 3 and day 7, suggesting that those gels are enriched with intermediate progenitor cells (Fig. 4F). Immature neurons are known to express T-box brain factor 1 (*TBR1*), which encodes a transcription factor that influences cortical lamination and is generally thought to directly follow TBR2 (*59*). While *TBR1* expression steadily increased in the DYN gels throughout the 7-day culture time, there was a reproducible and unexpected spike at day 3 in cells within Static gels that decreased again by day 7, implying a transient up-regulation in early neuronal cell fate within Static gels (Fig. 4G). Another common marker for early immature neurons is *TUBB3*, which encodes a microtubule element of the β-tubulin protein family that influences neurogenesis and axon guidance (*60*). The relative abundance of *TUBB3* was significantly increased in cells within both DYN gels compared to the Static gels (Fig. 4H). While the cells within DYN Fast gels were forming longer, more complex neurite networks compared to both DYN Slow and Static gels (Fig. 2, F to I), there were no significant differences in *TUBB3* expression between the two DYN gels.

Lastly, mature neurons are characterized by the expression of microtubule-associated protein 2 (MAP2), which stabilizes microtubules in the dendrites, and RNA binding fox-1 homolog 3 (RBFOX3), a neural-specific pre-mRNA alternative splicing regulator that also produces the widely used neuronal nuclei antigen (*61*, *62*). As with the expression of *TUBB3*, the expression of both *MAP2* and *RBFOX3* was significantly increased in cells encapsulated within both DYN gels compared to those in Static gels (Fig. 4, I and J). The expression of *MAP2* and *RBFOX3* was significantly higher in DYN Fast cells at day 7 compared to DYN Slow, suggesting an increase in neuronal maturation with increased stress relaxation rate.

To confirm hydrogel-mediated changes in gene expression at the protein-level, we measured the protein expression of several canonical neural lineage markers with Western blotting (fig. S12, A and B). At day 7 post-encapsulation, we observed the same trends as depicted by the gene expression data. The neuroepithelial cell marker, SOX2, was down-regulated in the DYN Fast gels compared to the Static gels (fig. S12C). Concurrently, we observed an up-regulation in the expression of immature and mature neuronal markers, TUBB3 and MAP2, respectively (fig. S12, D and E).

Together, these targeted mRNA and protein expression data suggest that hNPC cell fate acquisition is influenced by the relaxation rate of their microenvironment. In particular, hNPCs encapsulated within nonstress-relaxing, static hydrogels adopt expression profiles indicative of intermediate progenitors, while hNPCs encapsulated within stress-relaxing, dynamic hydrogels adopt more mature neuronal expression profiles. Moreover, as the matrix stress relaxation rate becomes faster, the proclivity for encapsulated cells to express markers of postmitotic neurons increases.

### Actin polymerization is required for hNPC neurite outgrowth and neural maturation in stress-relaxing matrices

**Fig. 5.**
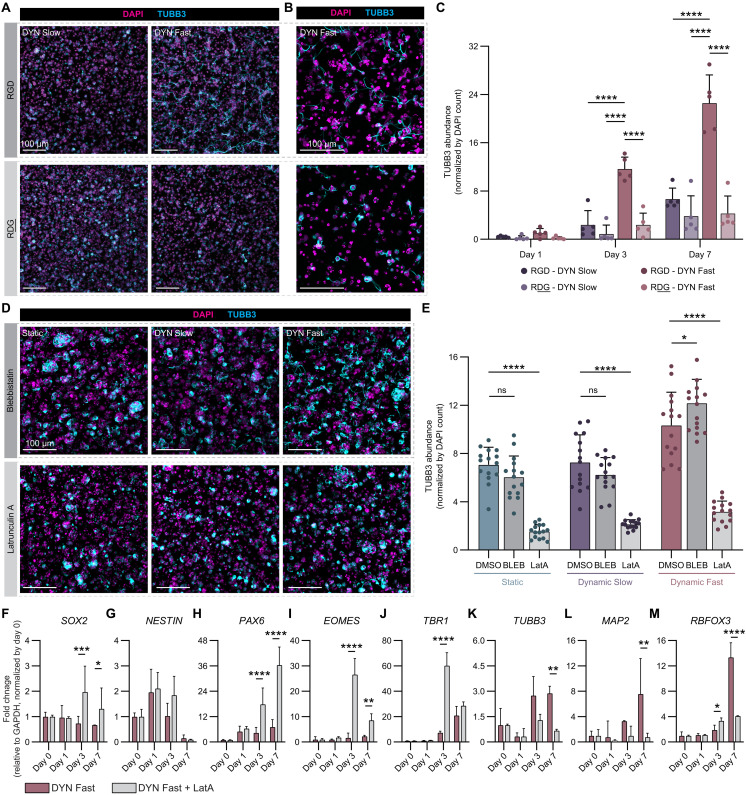
Actin polymerization is required for neurite outgrowth and neural maturation in stress-relaxing matrices. (**A**) Representative maximum projection fluorescence images of hNPC neuritic extension (DAPI-labeled nuclei in magenta, TUBB3-labeled neurites in cyan) after 7 days in culture (initially seeded at 3 × 10^4^ cells/μl) within DYN Slow and DYN Fast with either the cell-binding RGD amino acid sequence (top row) or the scrambled RDG sequence (bottom row) within the cell-interactive domain of the ELP. (**B**) Higher-magnification representative maximum projection fluorescence images of neuritic extension after 7 days in culture within DYN Slow and DYN Fast with either the cell-binding RGD or the scrambled RDG sequence. (**C**) Quantification of TUBB3-expressing neurite area, normalized by cell number, over time across DYN Slow and DYN Fast within either RGD- or RDG-containing matrices (*N* = 5 replicate hydrogels). (**D**) Representative maximum projection fluorescence images of neuritic extension after 7 days in culture within all three gel formulations following daily treatment with either blebbistatin (BLEB) or latrunculin A (LatA). (**E**) Quantification of TUBB3-expressing neurite area, normalized by cell number, over time across all three gel formulations following daily treatment with either blebbistatin or latrunculin A (*N* = 3 replicate hydrogels, *n* = 5 fields of view). (**F** to **M**) mRNA expression of canonical neural differentiation markers for hNPCs encapsulated in DYN Fast following treatment with latrunculin A over 7 days in culture (*N* = 4 replicate hydrogels). Statistical analyses performed as two-way ANOVA with Tukey’s multiple comparisons test (C), two-way ANOVA with Dunnett’s multiple comparisons test (E), and two-way ANOVA with Šídák’s multiple comparisons test (F to M). Data plotted as mean ± SD where *P < 0.05, **P < 0.01, ***P < 0.001, and ****P < 0.0001.

To interrogate the interactions that may link matrix relaxation rate to cell morphology, we began by characterizing the role of hNPC integrin binding within HELP. Integrin-mediated matrix adhesion is known to play a role in mechanosignaling; in particular, previous work from our lab found that the integrin-binding RGD ligand in the ELP biopolymer influenced neurite outgrowth in Schwann cells obtained from the dorsal root ganglia of chick embryos (*63*). To investigate the role of RGD signaling on hNPC morphology, we modified the amino acid sequence of the recombinant ELP protein to present a scrambled, cell-inert RDG sequence to which integrins cannot bind (*31*). We observed that hNPCs encapsulated in DYN Fast RDG matrices did not extend extensive neurites and exhibited morphologies similar to those encapsulated in DYN Slow matrices (Fig. 5, A and B). Despite being presented with a fast stress-relaxing microenvironment, the cells were observed to have significantly lower levels of elongated TUBB3-expressing neurites in the absence of RGD ligands (Fig. 5C). These results reveal that RGD-initiated integrin signaling is required for hNPCs to sense differences in matrix stress relaxation rate, which is necessary for mechanosensitive neurite outgrowth.

Given the role of RGD signaling on neurite outgrowth, we then investigated the mechanisms connecting hNPC neurite outgrowth with cell fate acquisition within stress-relaxing matrices. Previous work has implicated both cytoskeletal assembly and integrin signaling in mechanosensitive neural lineage commitment (*9*, *21*). Moreover, the transcriptomic data obtained from encapsulated hNPCs at day 7 suggested that matrix stress relaxation rate was influencing neural cytoskeletal dynamics (Fig. 3, C to H). To explore how cytoskeletal tension regulates neurite outgrowth, encapsulated hNPCs were exposed to small-molecule inhibitors of myosin II and actin polymerization (Fig. 5D). Neither inhibition resulted in differences in hNPC viability (fig. S13). Inhibition of myosin II by blebbistatin did not substantially alter the morphology of hNPCs or normalized TUBB3 abundance in Static and DYN Slow gels (Fig. 5, D and E). However, consistent with previous studies, slightly increased TUBB3 abundance was observed upon inhibition of actomyosin contractility when the microenvironment is permissive to neurite outgrowth, as in the RGD-containing, DYN Fast gels (Fig. 5E) (*64*). When actin polymerization was inhibited by latrunculin A, neurite outgrowth was abrogated, and hNPCs appeared morphologically similar to those cultured in Static gels regardless of gel condition (Fig. 5D). Furthermore, normalized TUBB3 abundance was significantly lower when actin polymerization was inhibited (Fig. 5E). Neurites extend along actin filament-based protrusions, so preventing actin polymerization reasonably reduces neurite extension (*65*). Together, these data reveal that actin polymerization is required for hNPC neurite extension in matrices with faster stress relaxation rates.

Finally, we sought to understand how pharmacological inhibition of neurite outgrowth, as opposed to matrix-driven inhibition (Fig. 4), would influence neural cell fate acquisition. To characterize changes in hNPC fate, we carried out targeted transcriptomic studies of hNPCs encapsulated in DYN Fast gels in the presence or absence of latrunculin A at days 0, 1, 3, and 7. Markers indicative of an immature progenitor cell fate (*SOX2* and *NESTIN*) exhibit a downward trend over time in control DYN Fast gels but are up-regulated when actin polymerization is inhibited (Fig. 5, F to G). Similarly, *PAX6* expression is significantly increased in the presence of latrunculin A (Fig. 5H). The transient intermediate progenitor cell marker, *EOMES*, and the immature neuronal marker, *TBR1*, are also both significantly up-regulated when neurite extension is prevented (Fig. 5, I and J), similar to the transient up-regulation in early neuronal cell fate observed in Static gels (Fig. 4, F and G). Conversely, genes present in more mature neurons (*TUBB3*, *MAP2*, and *RBFOX3*) are significantly decreased when neurite extension is prevented, as compared to the untreated control (Fig. 5, K to M). Together, the transcriptomic profile of hNPCs where neurite outgrowth is prevented due to inhibition of actin polymerization follows the same trends as that of hNPCs cultured within Static gels, where neurite outgrowth is limited by matrix properties (Fig. 4).

These results suggest that actin polymerization plays a critical role in transducing matrix stress relaxation–mediated neural maturation. Specifically, hNPC fate acquisition is influenced by cell morphology and the actin cytoskeleton, which can be modulated by the matrix stress relaxation rate (Fig. 2). Regardless of whether cell morphology is perturbed pharmacologically or by materials-based methods, preventing neurite outgrowth results in expression profiles indicative of intermediate progenitors. In contrast, permitting neurite outgrowth results in expression profiles representative of more mature neurons. These results support a mechanism by which faster stress relaxation rates enhance actin cytoskeleton assembly and neurite outgrowth, leading to increased expression of more mature neuronal markers.

## DISCUSSION

Together, our findings reveal that matrix viscoelasticity influences hNPC neurite outgrowth and cell fate acquisition in a 3D microenvironment. By leveraging static and dynamic covalent bonds, we created a family of HELP gels spanning a range of stress relaxation rates. We demonstrated that viscoelastic HELP gels support the 3D culture of hNPCs and promote neurite extension in fast stress-relaxing environments. These changes in neurite length and complexity were accompanied by altered proliferative capacity and metabolic activity, suggestive of a more mature phenotype. Through unbiased RNA-seq analysis and targeted transcriptomic studies at different time points, we identified differences in gene expression profiles that imply changes in the differentiation rates of hNPCs encapsulated within different stress-relaxing microenvironments. Furthermore, neurite extension within these hydrogels was found to be regulated by actin polymerization. Through independent manipulation of both external biophysical signaling (i.e., matrix stress relaxation rate) and intracellular cytoskeletal regulation (i.e., inhibition of actin polymerization), we identified the ability to extend neurites as a crucial morphological cue guiding hNPC fate acquisition.

Biochemical and biophysical signaling cues drive neural maturation both in vivo and in vitro (*1*). Conventionally, the continued differentiation and maturation of hNPCs occur stochastically with time in permissive media (*43*) or are promoted through the addition of exogenous growth factors which serve as biochemical signaling cues (*66*). Here, hNPCs were maintained in culture medium devoid of any exogenous growth factors. Hence, the morphological, physiological, and transcriptomic differences observed in encapsulated NPCs can be attributed primarily to differences in the biophysical properties of their microenvironment. As we were able to maintain a constant stiffness and ligand density across gel formulations which share the same polymer composition, the stress relaxation rate of the microenvironment served as the sole variable biophysical cue and, consequently, the main driver of the differences in NPC maturation.

3D protein-engineered biomaterials offer a tunable, controlled platform for incorporating biochemical and biophysical ECM-derived signals in in vitro models of human neural development. Natural biomaterials, including collagen, fibrin, and reconstituted basement membranes, exhibit stress relaxation and contain natural cell-interactive components (*11*), and early studies using Matrigel highlighted the importance of cell-ECM interactions in affecting neural differentiation (*67*, *68*). However, the ability to independently tune the stress relaxation rate of these natural biomaterials without altering other properties (i.e., stiffness and adhesive ligand density) poses a challenge. Furthermore, harvested materials such as Matrigel suffer from large batch-to-batch variability (*69*). More recently, synthetic materials, including polyethylene glycol (PEG), polyacrylamide, and peptide amphiphile hydrogels, as well as semi-synthetic materials like alginate, have been used because they are well-defined and highly tunable systems, enabling carefully defined interrogation into how specific matrix properties influence cell fate (*16*, *21*, *70*–*75*). Previous work using viscoelastic alginate hydrogels revealed that hNPCs exhibit greater transcriptional differences in response to changes in stress relaxation rate compared to changes in hydrogel stiffness and cell-adhesive ligand concentration; however, the range of stiffness profiled was significantly higher than the stiffness of our HELP hydrogels used in the present manuscript, and no nonstress-relaxing alginate was included as a potential control (*16*). In addition, alginate is naturally cell-inert, and so it must be modified with cell-adhesive ligands to promote bioactive signaling and adhesion before cell encapsulation. The recombinant protein-based biomaterials used here are reproducible, cell-interactive, and facilitate the independent tuning of biochemical and biophysical properties across a range of brain-mimetic stiffness and, hence, are an advantageous 3D culture platform for hNPCs.

While several studies have implicated matrix stiffness and degradability in neural cell behavior and differentiation, these studies were performed using elastic matrices (*5*, *9*, *32*, *36*, *76*), and little is known about how viscoelastic matrices regulate human neural cells. Our results offer complementary findings in agreement with these previous studies which primarily evaluated matrix stiffness and degradability. Here, we contextualize our findings with several such studies. First, rat neural stem cells were shown to undergo mechanosensitive lineage commitment within 3D matrices due to stiffness-dependent changes in confining stress, with softer, less confining matrices leading to increased neurogenesis and stiffer, more confining matrices leading to increased glial differentiation (*9*). Similar to cells encapsulated within a soft 3D matrix, hNPCs within DYN Fast HELP gels will experience less confining stress compared to those in Static gels, and the hNPCs in DYN Fast HELP also acquire a more mature neuronal fate. These findings suggest that confinement may also play a role in neural lineage commitment in viscoelastic matrices at a constant stiffness. Second, a pair of studies identified matrix degradability as a key mediator in enhancing murine NPC differentiation capacity (*32*, *36*). In these studies, a critical amount of cell-secreted enzyme-mediated remodeling was necessary to enable NPC stemness maintenance and subsequent differentiation. Here, the secretion of matrix degrading enzymes was similar in all three HELP gel formulations (fig. S8), yet the dynamic covalent cross-links in both DYN gels allowed for degradation-independent remodeling. While the mechanisms responsible for matrix remodeling varied between this study and those cited, with respect to their differentiation capacity, hNPCs within DYN Fast HELP gels seem to resemble cells encapsulated within highly degradable matrices. It should be noted that cells within viscoelastic HELP also exhibited improved viability over protracted culture times of 4 weeks, implying both a link between NPC differentiation and viability and a role for matrix remodelability in hNPC survival. Lastly, a recent study explored the effects of viscoelastic matrices on rat PC12 cells and observed that they exhibited increased neurite sprouting and elongation within faster stress-relaxing hydrogels (*77*). In this study, we shed light on the impact of matrix viscoelasticity on human NPCs. Specifically, we find that matrix stress relaxation rate is a critical regulator of integrin-mediated actin polymerization and, subsequently, hNPC morphology and cell fate acquisition, but the underlying molecular mechanisms need to be further studied to fully understand mechanosensing. In particular, future studies could explore the quantitative relationship between the kinetics of neural maturation and faster matrix stress relaxation rates, such as those reported for postnatal rat brain (Fig. 1D).

The brain is spatiotemporally patterned with respect to both cell fates and viscoelastic properties. Through indentation measurements on murine brains, hippocampal tissue exhibited strain stiffening behavior and a lower damping ratio compared to tissue from the cerebellum (*18*). The hippocampal dentate gyrus is one of the primary locations of adult neural stem cells (*78*–*80*), supporting the idea that neural cell types across a range of maturities experience a range of viscoelastic environments in the brain. In addition, magnetic resonance elastography experiments revealed an age-related decline in the viscosity of human brain tissue, suggesting a temporal variance in viscoelastic properties (*17*); however, no studies have been reported for earlier developmental timelines. A combination of factors could contribute to these variations, including changes in ECM composition over time (*2*) as well as differences in viscoelastic properties of distinct cell types (*81*). Finer resolution rheological measurements would be required to further investigate the emerging link between spatiotemporal differences in brain viscoelasticity and the effect on neural maturation in vivo.

Another interesting finding is that cell morphology and actin cytoskeleton rearrangement were highly correlated with hNPC cell fate. We modulated hNPC morphology through two distinct methods: (i) enhancing neurite outgrowth by manipulating the material microenvironment and (ii) restricting neurite extension by pharmacological inhibition of actin polymerization. When presented with a stress-relaxing environment that is permissive to neurite outgrowth, morphological changes regulate the ability of hNPCs to acquire more mature neuronal cell fates. Conversely, restricting neurite outgrowth either by encapsulating hNPCs in a nonstress-relaxing environment or by treating encapsulated hNPCs with inhibitors of actin polymerization prevents further neural maturation. Both experiments suggest that hNPC morphology, namely, the ability to extend neurites, is critically linked to hNPC differentiation. Given that neurite outgrowth, neuritic branching, axon specification, and pathfinding are key morphological hallmarks of neuronal maturation in vivo, it follows that hNPC shape may be implicated in neural cell fate acquisition in vitro. In nonhuman cultures, actin polymerization has been implicated in several maturation-related neuronal processes including axon branching, growth cone motility, axon guidance, and axon retraction (*82*–*86*). Consistent with these observations from in vivo and in vitro nonhuman samples, hNPC morphology appears to be linked to cell fate through a mechanism that involves altered actin cytoarchitecture.

Overall, this work presents a hydrogel system where matrix stress relaxation rate can be specified independently of matrix stiffness and cell-adhesive ligand density. Encapsulation and 3D culture of hNPCs within these materials have revealed the role of matrix viscoelasticity in modulating neural maturation through effects on actin architecture. These 3D biomaterial design principles can be leveraged to study other facets of neural cell behavior in more advanced in vitro models of the neural microenvironment, including potential applications in drug discovery and disease modeling.

## MATERIALS AND METHODS

### ELP expression

Recombinant ELP was expressed and purified as previously described (*31*). Briefly, pET15b plasmids encoding the ELP sequence containing either the RGD or RDG motif under control of the T7 promoter were transformed into BL21(DE3)pLysS *Escherichia coli* (Invitrogen). *E. coli* were cultured in terrific broth until an optical density at 600 nm of 0.8 was reached, after which ELP expression was induced by 1 mM isopropyl β-d-1-thiogalactopyranoside (Thermo Fisher Scientific). After 7 hours, the cells were pelleted by centrifugation and lysed via alternating freeze-thaw cycles in TEN buffer [10 mM tris, 100 mM NaCl, and 1 mM EDTA, (pH 8.0)] supplemented with 10 μM deoxyribonuclease I (Sigma-Aldrich) and 1 mM phenylmethanesulfonyl fluoride (PMSF; MP Biomedicals) protease inhibitor. ELPs were purified through three rounds of thermal cycling, dialyzed against Milli-Q water for 3 days, lyophilized, and stored at −20°C.

### Synthesis and characterization of hydrazine-functionalized ELP

ELP was modified with hydrazine as previously described (*28*). Briefly, lyophilized ELP was dissolved in anhydrous dimethyl sulfoxide (DMSO; Sigma-Aldrich) to a concentration of 7.3 wt % at room temperature (RT). Once fully dissolved, an equal volume of anhydrous *N*,*N*-dimethylformamide (DMF; Sigma-Aldrich) was added. In a separate flask, tri-Boc hydrazinoacetic acid (2.1 eq. per ELP amine; Sigma-Aldrich) and hexafluorophosphate azabenzotriazole tetramethyl uronium (HATU; 2 eq. per ELP amine; Sigma-Aldrich) were dissolved in the same volume of DMF used to dissolve the ELP. Once dissolved, 4-methylmorpholine (5 eq. per ELP amine; Sigma-Aldrich) was quickly added and allowed to react for 10 min at RT. Next, the tri-Boc hydrazinoacetic acid solution was added to the ELP solution in a dropwise manner and left to react overnight at RT. The reaction was then precipitated in ice-cold diethyl ether (Thermo Fisher Scientific), pelleted, and dried under nitrogen gas to yield Boc-protected ELP. The Boc-protecting groups were removed via acid-mediated deprotection by dissolving the Boc-protected ELP in a 1:1 mixture of dichloromethane (DCM; Sigma-Aldrich) and trifluoroacetic acid (TFA; Sigma-Aldrich) supplemented with 5% (v/v) triisopropylsilane (Sigma-Aldrich) to a concentration of 3.3 wt %. The reaction was stirred for 4 hours at RT, then precipitated in ice-cold diethyl ether, pelleted, and dried under nitrogen gas. The resulting pellet was dissolved in ice-cold Milli-Q water, dialyzed against Milli-Q water for 3 days, sterile-filtered, lyophilized, and stored at −20°C.

To quantify the modification efficiency, samples before the removal of Boc groups were assessed by ^1^H nuclear magnetic resonance (NMR) spectroscopy (Varian Inova, 500 MHz) using deuterated DMSO (Thermo Fisher Scientific) as a solvent. The modification efficiency was determined by comparing the integration of methyl protons of the Boc group (δ = 1.46 and 1.39, 27H) with the aromatic protons of tyrosine (δ = 7.00 and 6.62, 4H) in Boc-protected ELP, and the modification calculation is shown in fig. S2. Removal of Boc groups was also verified by ^1^H NMR.

### Synthesis and characterization of norbornene-functionalized ELP

Before modifying the ELP with norbornene, the hydrophilicity of the protein was altered by conjugating a PEG oligomer via an amidation reaction. Lyophilized ELP was dissolved in anhydrous DMSO to a concentration of 7.5 wt % at RT. Once fully dissolved, an equal volume of anhydrous DMF was added. In a separate flask, t-Boc-*N*-amido-PEG12-acid (2 eq. per ELP amine, BroadPharm) and HATU (2.2 eq. per ELP amine) were dissolved in the same volume of DMF used to dissolve the ELP. Once dissolved, 4-methylmorpholine (5 eq. per ELP amine) was quickly added and allowed to react for 10 min at RT. Next, the t-Boc-*N*-amido-PEG12-acid solution was added to the ELP solution slowly over the course of 10 min and left to react overnight at RT. The reaction was then precipitated in ice-cold diethyl ether, pelleted, and dried under nitrogen gas. The Boc-protecting groups were removed via acid-mediated deprotection by dissolving the Boc-protected ELP in a 1:1 mixture of DCM and TFA supplemented with 5% (v/v) triisopropylsilane to a concentration of 3.3 wt %. The reaction was stirred for 4 hours at RT, then precipitated in ice-cold diethyl ether, pelleted, and dried under nitrogen gas. The resulting pellet was dissolved in ice-cold Milli-Q water, dialyzed against Milli-Q water for 3 days, and lyophilized.

The conjugation of norbornene was performed via a subsequent HATU-mediated amidation reaction. The PEG-modified ELP was dissolved in anhydrous DMSO at 7.5 wt % at RT. Once fully dissolved, an equal volume of anhydrous DMF was added. In a separate flask, exo-5-norbornene carboxylic acid (2 eq. per ELP amine; Sigma-Aldrich) and HATU (2.2 eq. per ELP amine) were dissolved in the same volume of DMF used to dissolve the ELP. Once dissolved, 4-methylmorpholine (5 eq. per ELP amine) was quickly added and allowed to react for 10 min at RT. Next, the exo-5-norbornene carboxylic acid solution was added to the ELP solution slowly over the course of 10 min and left to react overnight at RT. The reaction was then precipitated in ice-cold diethyl ether, pelleted, and dried under nitrogen gas. The resulting pellet was dissolved in ice-cold Milli-Q water, dialyzed against Milli-Q water for 3 days, sterile-filtered, lyophilized, and stored at −20°C.

To quantify the modification efficiency, samples were assessed by ^1^H NMR spectroscopy using deuterated water (Thermo Fisher Scientific) as a solvent. The modification efficiency was determined by integration of the proton signal of the alkene hydrogens on the norbornene ring (δ = 6.2, 2H) relative to the protons of the four available tyrosine peaks (δ = 7.25 and 6.75, 4H).

### Synthesis and characterization of benzaldehyde- and aldehyde-modified HA

In the first step of HA-benzaldehyde (BZA) or HA-aldehyde (ALD) synthesis, 100-kDa sodium hyaluronate (HA; LifeCore Biomedical) undergoes a 1-ethyl-3-(3-dimethylaminopropyl)carbodiimide hydrochloride (EDC) carbodiimide cross-linking reaction to form an HA-alkyne intermediate polymer, as described previously (*28*). Briefly, HA was dissolved in 2-(N-morpholino)ethanesulfonic acid (MES) buffer [0.2 M MES hydrate (Sigma-Aldrich) and 0.15 M NaCl in Milli-Q water (pH 4.5)] at 1 wt %. Once fully dissolved, propargylamine (0.8 eq. per HA dimer unit; Sigma-Aldrich) was added, and the pH was immediately adjusted to 6.0 using 1 N NaOH. *N*-hydroxysuccinimide (0.8 eq. per HA dimer unit; Thermo Fisher Scientific) and EDC (0.8 eq. per HA dimer unit; Thermo Fisher Scientific) were added sequentially. The reaction was allowed to stir overnight at RT. The reaction was then dialyzed against Milli-Q water for 3 days, sterile-filtered, lyophilized, and stored at −20°C.

The conjugation of benzaldehyde or aldehyde was performed via subsequent copper-catalyzed azide-alkyne click chemistry of either a small-molecule azidobenzaldehyde [synthesis previously described (*87*)] or Ald-CH2-PEG3-azide (BroadPharm) to achieve HA-BZA or HA-ALD, respectively. Lyophilized HA-alkyne was dissolved in 10× phosphate-buffered saline [PBS; 81 mM sodium phosphate dibasic, 19 mM sodium phosphate monobasic, and 60 mM sodium chloride in Milli-Q water (pH 7.4)] supplemented with β-cyclodextrin (1 mg/ml; Sigma-Aldrich) at 1 wt %. Once fully dissolved, the solution was degassed under nitrogen for 30 min. Solutions of sodium ascorbate (0.18 eq. per HA dimer unit, Sigma-Aldrich) and copper (II) sulfate pentahydrate (0.0096 eq. per HA dimer unit, Sigma-Aldrich) dissolved in Milli-Q water were also degassed under nitrogen and sequentially added to the HA-alkyne solution. For HA-BZA, synthesized azidobenzaldehyde (2 eq. per alkyne group) dissolved in anhydrous DMSO was added to the HA-alkyne solution and degassed for an additional 10 min. For HA-ALD, Ald-CH2-PEG3-azide (2 eq. per alkyne group) was directly added to the HA-alkyne solution and degassed for an additional 10 min. The reaction was allowed to stir for 24 hours at RT, and then an equal volume of 50 mM EDTA (pH 7.0; Thermo Fisher Scientific) was added to chelate the copper for 1 hour. The reaction was then dialyzed against Milli-Q water for 3 days, sterile-filtered, lyophilized, and stored at −20°C.

To quantify the modification efficiency, samples were assessed by ^1^H NMR spectroscopy using deuterated water as a solvent. The modification efficiency of HA-benzaldehyde was determined by measuring the protons on the benzene ring (δ = 7.93 and 7.82, 4H), triazole linkage (δ = 7.9, 1H), and aldehyde group (δ = 9.9, 1H) relative to the protons of the acetyl group (δ = 1.8, 3H). The modification efficiency of HA-aldehyde was determined by measuring the protons on the triazole linkage (δ = 7.9, 1H) relative to the protons of the acetyl group (δ = 1.8, 3H).

### Synthesis and characterization of tetrazine-modified HA

HA (100 kDa) was modified with tetrazine in a similar amidation reaction as previously described (*88*). HA was dissolved in 0.1 M MES buffer (pH 7) at 1 wt %. Once fully dissolved, 1-hydrozybenzotriazole hydrate (2 eq. per HA dimer unit; Sigma-Aldrich) was added to the HA and allowed to dissolve for 15 min. In a separate flask, tetrazine amine (2 eq. per HA dimer unit; Conju-Probe) was dissolved in a 5:1 mixture of acetonitrile (MeCN; Sigma-Aldrich) and deionized water. Once dissolved, EDC (2 eq. per HA dimer unit) was added and allowed to dissolve. The solution was then added over the course of 30 min into the dissolved HA solution and allowed to react overnight at RT. The reaction mixture was dialyzed for 2 days against a 10% MeCN solution, followed by 3 days against Milli-Q water. The solution was then sterile-filtered, lyophilized, and stored at −20°C.

To quantify the modification efficiency, samples were assessed by ^1^H NMR spectroscopy using deuterated water as a solvent. The modification efficiency of HA-tetrazine was determined by measuring the protons on the tyrosine peaks (δ = 8.5 and 7.6, 4H) and tetrazine peak (δ = 10.3, 1H) relative to the protons of the acetyl group (δ = 1.8, 3H).

### Formation of HELP hydrogels

Lyophilized ELP and HA components were dissolved in 10× PBS [81 mM sodium phosphate dibasic, 19 mM sodium phosphate monobasic, and 60 mM sodium chloride in Milli-Q water (pH 7.4)] at a stock concentration of 2 wt % overnight at 4°C. The final solutions were kept on ice until use. For Static HELP gels, an equal volume of 2 wt % ELP and HA solutions were mixed in a tube, and 10 μl of the resulting mixture was pipetted into each silicone mold (4 mm in diameter, 0.8 mm in height, plasma bonded to a 12-mm circular #2 coverglass, Electron Microscopy Sciences). The gels were immediately incubated on ice for 5 min, followed by RT for 15 min, and lastly 37°C for 10 min. For DYN HELP gels, 5 μl of 2 wt % HA solution was added to each silicone mold, and an equal volume of 2 wt % ELP solution was pipetted directly onto the HA. The two components were immediately mixed using the same pipette tip. Following mixing, the gels were immediately incubated at RT for 15 min followed by 37°C for 10 min.

### Hydrogel rheology

Mechanical testing was carried out using a stress-controlled ARG2 rheometer (TA Instruments) using a cone-on-plate geometry (20 mm in diameter, 1° cone angle). Hydrogel samples of 48 μl were deposited onto the rheometer stage, and heavy mineral oil was used to fill the gap in between the geometry and the external environment to keep the sample hydrated. DYN HELP gels were allowed to cross-link under 1% oscillatory strain and 1 rad/s angular frequency for 15 min at 23°C followed by 15 min at 37°C. Static HELP gels were allowed to cross-link under 1% oscillatory strain and 1 rad/s angular frequency for 5 min at 4°C followed by 15 min at 23°C and 15 min at 37°C. To evaluate the elastic properties of our hydrogels, a frequency sweep from 0.1 to 100 rad/s was then performed under 1% oscillatory strain at 37°C. The storage and loss moduli were taken to be the values at 1 rad/s from these measurements. Following the frequency sweep, the samples were incubated under 1% oscillatory strain and 1 rad/s for 5 min at 37°C. Given the angular frequency limits of our rheometer, we characterized the viscous properties of our hydrogels using a stress relaxation test. Stress relaxation tests were performed by straining each sample to 10% and measuring the stress over a period of 24 hours.

### Fluorescence recovery after photobleaching

FRAP was performed to measure HELP hydrogel diffusivity as previously described (*89*). Briefly, 30 μl of gels was formed at the bottom of a clear-bottom, half-area, black 96-well plate (Greiner Bio-One). Once gels had formed, solutions of fluorescein isothiocyanate–labeled dextrans (4 mg/ml; Sigma-Aldrich) of varying molecular weights (10, 20, 40, 70, 150, and 250 k) were added to each respective well and allowed to incubate at 37°C overnight. Using a confocal microscope (Leica SPE), a 100 μm by 100 μm area of each hydrogel was photobleached using a 488-nm laser at 100% intensity for 1 min. Immediately following photobleaching, fluorescence recovery into the photobleached region was monitored for 4 min (1 frame/s) at 10% laser intensity. Images were analyzed using open source MATLAB code that was previously published (*90*).

### Murine brain rheology

Sprague-Dawley rats (female, 24 weeks of age, RNU^−/−^ athymic, Charles River Laboratories) were euthanized in compliance with National Institutes of Health and institutional guidelines. Full brains were collected immediately after euthanization and placed in Dulbecco's phosphate-buffered saline (DPBS) on ice. Mechanical measurements were collected between 1 to 2 hours following resection. Each tissue was trimmed using a biopsy punch and razor blade into 8-mm-diameter and 2- to 4-mm-thick sections and allowed to come to RT in DPBS for 30 min. As previously described, mechanical characterization was performed using a stress-controlled ARG2 rheometer with a parallel plate geometry (8 mm) (*87*). To prevent the tissues from slipping, both the rheometer stage and geometry head were affixed with a thin section of sandpaper. Storage moduli and stress relaxation data were obtained as described above with the caveat that measurements were only taken once the normal force reached a value of 0.1 to 0.2 N. All measurements were collected at RT.

### hiPSC maintenance

All hiPSCs were previously validated with respect to their stemness and differentiation capacity (*91*). The hiPSCs were routinely tested for mycoplasma. In total, four hiPSC lines from two donors were included. Approval for this study was obtained from the Stanford Institutional Review Board, and informed consent was obtained from all donors. hiPSCs were maintained in their pluripotent state by being cultured with mTESR-1 Plus (STEMCELL Technologies) media in monolayer on human embroyonic stem cell (hESC)–qualified Matrigel (0.1 mg/ml; Sigma-Aldrich).

### NPC differentiation

hiPSCs were differentiated into hNPCs with a commercially available dual SMAD inhibition kit (STEMCELL Technologies). hiPSCs were expanded to approximately 95% confluency on hESC-qualified Matrigel (Sigma-Aldrich) before exposure to the provided STEMdiff Neural Induction Medium. Daily media changes with the induction medium were performed for 10 days. On day 11, cells were dissociated with cell dissociation solution (Sigma-Aldrich), passaged onto poly-d-lysine (50 μg/ml; Sigma-Aldrich) and laminin (10 μg/ml; Roche)–coated plates. From day 11 to days 18 to 22, the resultant hNPCs were maintained in N3 culture medium consisting of DMEM/F12 (Thermo Fisher Scientific), neurobasal (Thermo Fisher Scientific), N-2 supplement (1%, Thermo Fisher Scientific), B-27 supplement with vitamin A (2%, Thermo Fisher Scientific), GlutaMax (1%, Thermo Fisher Scientific), and Minimal Essential Medium Nonessential Amino Acids (1%, Thermo Fisher Scientific) in accordance with previously published protocols (*43*, *92*).

### NPC encapsulation

Before encapsulation, cells were dissociated, filtered through a 70-μm cell strainer, pelleted by centrifugation, and counted. Cell pellets were resuspended in 2 wt % ELP solution at 2× the desired final cell density (i.e., 6 × 10^4^ cells/μl for a final cell density of 3 × 10^4^ cells/μl). The cell suspensions were mixed thoroughly with a p200 pipette. HELP matrix formation with cells was performed as described above, and then 1 ml of prewarmed N3 media was added to each well of a 24-well plate, which contained one 10-μl hydrogel. Medium was replenished daily.

### Cellular viability

To characterize viability, hNPC-laden hydrogels were cultured in a solution of DPBS supplemented with 2 μM calcein acetoxymethyl and 4 μM ethidium homodimer for 20 min at 37°C. The gels were washed with DPBS and imaged with a confocal microscope (Leica SPE).

### Cellular proliferation and metabolism

To characterize proliferation and metabolism, dsDNA was quantified and used to normalize the metabolic capacity of encapsulated hNPCs. Following the manufacturer’s instructions, the Quant-iT Picogreen kit (Thermo Fisher Scientific) was used to measure dsDNA across days in culture. All samples were run in triplicate. Then, the metabolic capacity of the cells was quantified following the manufacturer’s instructions with the CellTiter-Blue kit (Promega). Metabolic data from a given gel were normalized by the amount of dsDNA from that gel.

### Cell-secreted enzyme activity

To characterize hyaluronidase and protease activity, hNPC-laden gels were assayed using two different kits: the Hyaluronidase Assay Kit (Amsbio, Ra003-01-HAK) and the ProteSEEKER protease screening assay (G-Biosciences, 786-325), respectively. Each kit was used according to the manufacturer’s instructions.

### Immunocytochemistry

hNPC-laden hydrogels were washed with DPBS and fixed with 4% paraformaldehyde in DPBS for 30 min at 37°C. Samples were permeabilized for 1 hour at RT with 0.25% (v/v) Triton X-100 in PBS (PBST) and blocked for 3 hours at RT in PBS with 5% (w/v) bovine serum albumin (BSA; Roche), 5% (v/v) goat serum (Gibco), and 0.5% (v/v) Triton X-100. Primary antibody dilutions were prepared in PBS with 2.5% (w/v) BSA, 2.5% (v/v) goat serum, and 0.5% (v/v) Triton X-100 (antibody dilution solution). Primary antibodies were diluted as follows: rat anti-Ki67 (1:100; Thermo Fisher Scientific, 14-5698-82), mouse anti-pHH3 (1:200; Santa Cruz, sc-374669), and mouse anti–β3-tubulin (1:200; Cell Signaling, 4466). Following incubation with primary antibodies overnight at 4°C, the hydrogels were washed with PBST three times for 30 min per wash. Secondary antibodies were diluted in antibody dilution solution as follows: goat anti-rat Alexa Fluor 488 (1:500; Invitrogen, no. A-11006), goat anti-mouse Alexa Fluor 488 (1:500; Invitrogen, no. A-11001), goat anti-mouse Alexa Fluor 546 (1:500; Invitrogen, no. A-11030), and goat anti-mouse Alexa Fluor 647 (1:500; Invitrogen, no. A-21235). 4′,6-Diamidino-2-phenylindole (DAPI; 1 μg/ml; Cell Signaling, 4083s) was included in the secondary antibody solution to stain nuclei. Samples were incubated with the second antibody solution overnight at 4°C before being washed with PBST three times for 30 min per wash. The resultant stained hydrogels were mounted onto no. 1 coverslips with ProLong Gold Antifade Mountant (Thermo Fisher Scientific) and allowed to cure for 48 hours before imaging with a confocal microscope (Leica SPE). To avoid any possible confounds imparted by the mechanical properties of the glass, all images were taken at a *z*-depth at least 50 μm from the coverslips.

### Automated image analysis

The number of DAPI^+^ nuclei, pHH3^+^ cells, and Ki67^+^ cells and the area occupied by TUBB3^+^ neurites were quantified with automated scripts written in CellProfiler (*93*). Briefly, nuclei and cells which were immunolabeled with pHH3 and Ki67 were identified using “IdentifyPrimaryObjects” with the “Minimum Cross-Entropy” thresholding method. TUBB3-labeled neurites were identified by passing the TUBB3 images through the following series of steps: “EnhanceOrSuppressFeatures,” “RescaleIntensity,” “IdentifyPrimaryObjects,” and “MeasureImageAreaOccupied.” The minimum diameter of the neurite objects was adjusted such that only neuritic projects which extended a specific distance away from the center of the object, herein set to 18 pixels, were counted.

### Manual neurite tracing

To characterize the length and branching complexity of individual neurites, TUBB3-expressing projections were manually traced and quantified with the SNT toolbox (*94*). Neurites with a total length less than 5 μm were not traced; otherwise, to the greatest degree possible, all neurites longer than 5 μm were traced in 3D space.

### RNA-seq and data analysis

Agnostic quantification of mRNA expression was achieved with 3′ RNA-seq. Frozen hydrogel pellets were submitted to Lexogen for several stages of the sequencing and analysis process including sample homogenization with the 1600 MiniG (1400 rpm for 30 s), mRNA extraction with the SPLIT RNA Extraction Kit, library preparation with the QuantSeq-Pool Sample-Barcoded 3′ mRNA-Seq Library Prep Kit, 3′ RNA-seq, demultiplexing with idemux, unique molecular identifier extraction with umi_tools (v1.0.1), read trimming with cutadapt (v2.5), read alignment to hg38 with STAR aligner (v2.7.5a), deduplication with umi_tools (v1.0.1), and alignment counting with featureCounts (subread v1.6.4). The QuantSeq workflow is available for download through Lexogen’s public-facing GitHub: https://github.com/Lexogen-Tools/quantseqpool_analysis. Downstream analysis of the provided count data, including normalization and differential expression analysis, was performed with DESeq2 (v3.17) and associated Bioconductor packages in R-4.2.2.

### Quantitative reverse transcription polymerase chain reaction

Targeted quantification of mRNA expression was achieved with quantitative reverse transcription polymerase chain reaction. First, to release encapsulated cells from HELP, the hydrogels were transferred to Eppendorf tubes and treated with 10 μl of a solution of DPBS supplemented with elastase (250 U/ml; GoldBio), hyaluronidase (2000 U/ml; Sigma-Aldrich), and 3 mM EDTA (Thermo Fisher Scientific) for 30 min at 37°C. The samples were then resuspended in 500 μl of TRIzol reagent (Invitrogen) and immediately transferred to a −80°C freezer until use. The samples were thawed on ice and disrupted with probe sonication [Heilscher UP50H, 50% amplitude (25 W), 30-kHz frequency, 0.5-s cycle]. mRNA was purified with a phenol-chloroform extraction using 5PRIME phase lock gels (Quantabio) and subsequent isopropyl alcohol precipitation. The mRNA concentration of resuspended solutions was measured with a NanoDrop (Thermo Fisher Scientific), and 100 ng of mRNA was reverse-transcribed per sample with the High-Capacity cDNA Reverse Transcription Kit (Applied Biosystems). The resultant cDNA (6.6 μl) was mixed with 0.9 μl of a 5 μM solution of forward and reverse primer pairs (Integrated DNA Technologies; table S1) and 7.5 μl of Fast SYBR Green Master Mix (Applied Biosystems). Quantitative polymerase chain reaction was performed with the StepOnePlus Real Time PCR System (Applied Biosystems). Cycle threshold (CT) values were calculated using the StepOnePlus software (v.2.3) and analyzed by the ΔCT method. Statistical analysis was performed before transforming to a natural scale, and relative mRNA expression is reported as a geometric mean with 95% confidence intervals derived from the nontransformed data.

### Western blot

hNPCs were encapsulated within 20 μl of HELP as described above at an initial concentration of 3 × 10^4^ cells/μl. To release encapsulated cells from HELP, the hydrogels were transferred to Eppendorf tubes and treated with 20 μl of a solution of DPBS supplemented with elastase (250 U/ml; GoldBio), hyaluronidase (2000 U/ml; Sigma-Aldrich), and 3 mM EDTA (Thermo Fisher Scientific) for 30 min at 37°C. The samples were then resuspended in 100 μl of radioimmunoprecipitation assay lysis buffer solution supplemented with 1 mM PMSF and protease inhibitor tablets (Roche), incubated on ice for 20 min, and frozen at −80°C until use. Samples were thawed on ice, and 20 μl of lysate was combined with 5 μl of 5× Laemmli buffer [50% (v/v) glycerol, 10 wt % sodium dodecyl sulfate, 0.05 wt % bromophenol blue, and 300 mM tris (pH 6.8)] supplemented with fresh 500 mM dithiothreitol. Samples were incubated at 96°C for 10 min to denature the protein and then loaded into precast polyacrylamide gels (Bio-Rad) along with a protein ladder (Bio-Rad). The gel was run at 140 V for 90 min and transferred to a methanol-activated polyvinylidene fluoride membrane (Invitrogen) via wet transfer for 1 hour at 100 V. The membrane was cut to enable staining of different molecular weight proteins from the same gel and blocked for 1 hour in blocking solution: 5 wt % milk in tris-buffered saline [20× stock: 3 M NaCl and 750 mM tris hydrochloride (pH 7.2)] supplemented with 0.25% (v/v) Tween-20 (TBST). Primary antibodies were diluted in blocking solution as follows: rabbit anti-SOX2 (1:1000; Cell Signaling, 23064), chicken anti–β3-tubulin (1:1000; Aves Labs, TUJ), and rabbit anti-MAP2 (1:1000; Cell Signaling, 4542). Samples were incubated overnight rocking at 4°C. The next day, membranes were washed in TBST three times for 5 min per wash. Horseradish peroxidase–conjugated secondary antibodies were diluted in TBST as follows: HRP-donkey–anti-rabbit (1:10,000; Jackson ImmunoResearch, 711-035-152) and HRP-donkey–anti-chicken (1:10,000; Jackson ImmunoResearch, 703-035-155). Samples were incubated for 1 hour while rocking at RT before being washed with TBST three times for 10 min per wash. The blot was developed using either the SuperSignal West Pico or Femto Chemiluminescent Substrate (Thermo Fisher Scientific) and imaged using a ChemiDoc MP gel imaging system (Bio-Rad). Densitometry analysis was performed using ImageJ to quantify the blots in fig. S12.

### Cytoskeleton inhibitors

To inhibit actin polymerization and myosin II, latrunculin A (Tocris) was used at 1 μM, and blebbistatin (Abcam) was used at 10 μM, respectively. Inhibitors were added to the appropriate gels on day 1 and replenished daily. Gels were fixed on day 7 for staining, and TUBB3 abundance was quantified, as described above.

### Statistical analysis and reproducibility

The data collected for this manuscript were obtained from four different hiPSC lines obtained from two donors, five distinct rounds of hiPSC to NPC differentiation, well over 30 distinct batches of NPC thawing to initiate encapsulated NPC cultures, two expressions and modifications of ELP, and four modifications of HA. Results were consistent across all biological and material batches. Statistical analyses were performed using GraphPad Prism v.9.3.1. Details of specific statistical methods, *P* value results, biological cell lines tested, and biological replicates (i.e., number of times an experiment was independently performed), and technical replicates are summarized in table S2. For all studies, not significant (ns; *P* > 0.05), *P < 0.05, **P < 0.01, ***P < 0.001, and ****P < 0.0001.
