# Supplementary material for: Tunable hydrogel viscoelasticity modulates human neural maturation
Source: Sci Adv. 2023 Oct 20;9(42):eadh8313. doi: 10.1126/sciadv.adh8313 (PMC10588948; doi:10.1126/sciadv.adh8313)
Supplement: Supplementary file 1 — Figs. S1 to S13 Table S1 Legend for table S2 [file sciadv.adh8313_sm.pdf]

Science Advances
AAAS

Supplementary Materials for

Tunable hydrogel viscoelasticity modulates human neural maturation

Julien G. Roth *et al.*

Corresponding author: Sarah C. Heilshorn, heilshorn@stanford.edu

Sci. Adv. **9**, eadh8313 (2023)
DOI: 10.1126/sciadv.adh8313

The PDF file includes:

Figs. S1 to S13
Table S1
Legend for table S2

Other Supplementary Material for this manuscript includes the following:

Table S2

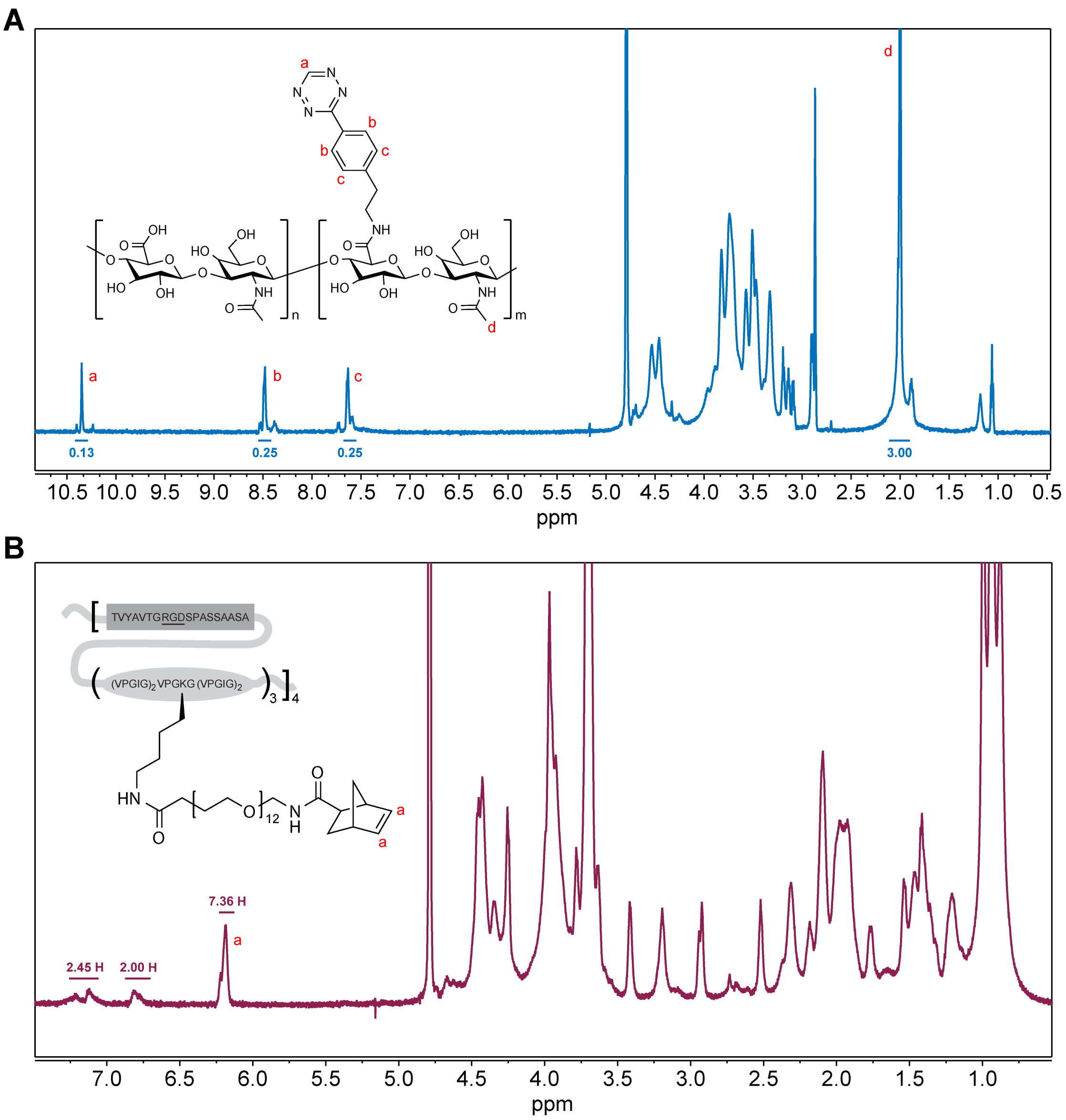

Fig. S1. Hyaluronan (HA) modified with tetrazine functional groups and elastin-like protein (ELP) modified with norbornene functional groups.

A. *Representative proton nuclear magnetic resonance (^{1}H NMR; D_2O solvent) spectra of 13% modified HA-tetrazine polymer. The modification efficiency was determined by measuring the protons on the tyrosine peaks (δ = 8.5 and 7.6, 4H) and tetrazine peak (δ = 10.3, 1H) relative to the protons of the acetyl group (δ = 1.8, 3H).* ***B.*** *Representative ^{1}H NMR (D_2O solvent) spectra of 94.5% modified ELP-norbornene polymer. The modification efficiency was determined by integration of the proton signal of the alkene hydrogens on the norbornene ring (δ = 6.2, 2H) relative to the protons of the four available tyrosine peaks (δ = 7.25 and 6.75, 4H).*

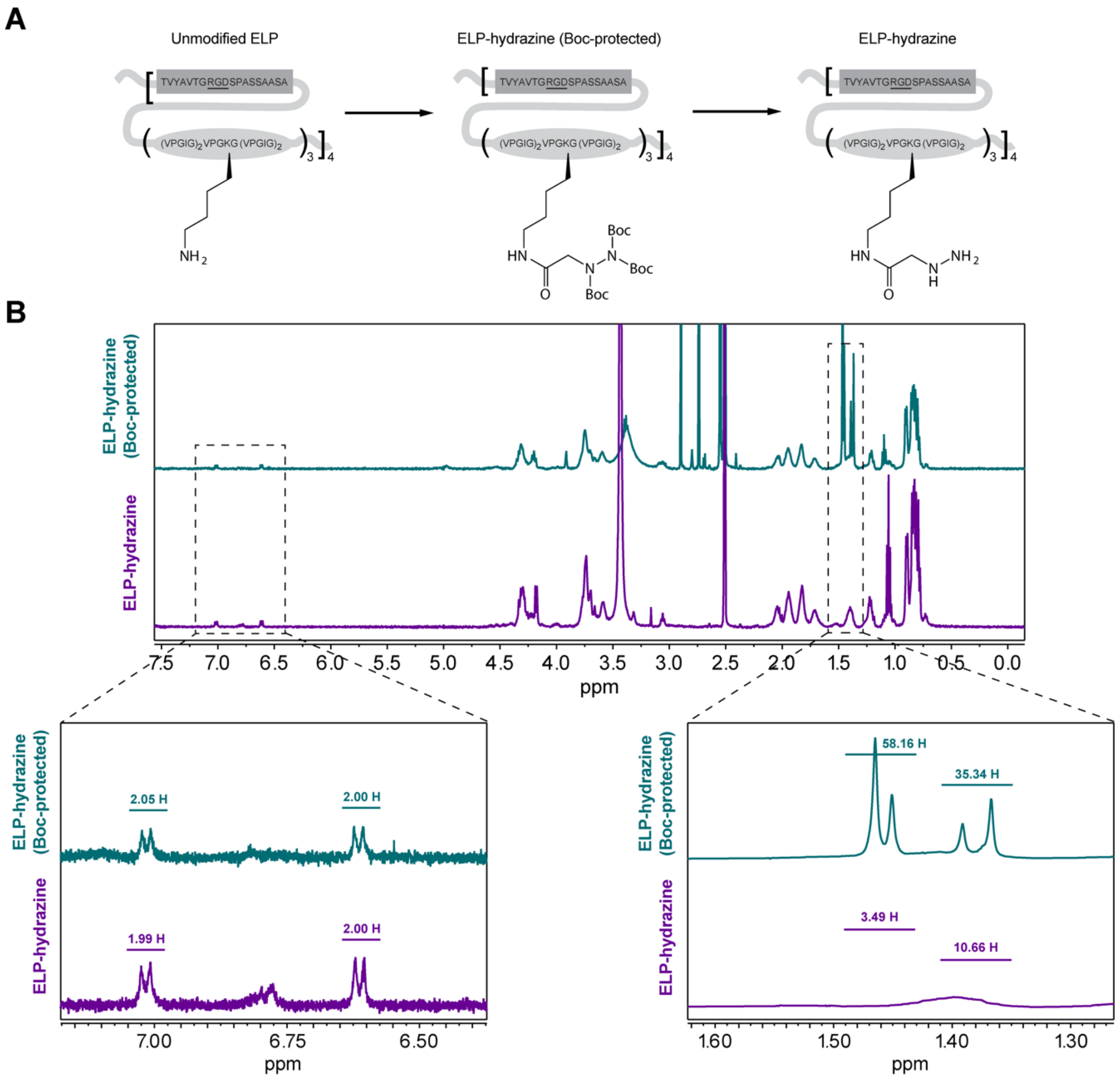

C

$$\frac{Hydrazine\ groups}{ELP} = \frac{Boc\ peak\ H}{tyrosine\ peak\ H} \times \frac{4\ H}{tyrosine} \times \frac{4\ tyrosine}{ELP} \times \frac{1\ Boc}{9\ H} \times \frac{1\ hydrazine}{3\ Boc}$$

$$\frac{Hydrazine\ groups}{ELP} = \frac{93.5\ H}{4.05\ H} \times \frac{4\ H}{tyrosine} \times \frac{4\ tyrosine}{ELP} \times \frac{1\ Boc}{9\ H} \times \frac{1\ hydrazine}{3\ Boc} = 13.68$$

$$\frac{13.68}{14\ possible\ hydrazine\ groups} = \sim 97.7\%\ modification\ of\ ELP$$

Fig. S2. Elastin-like protein (ELP) modified with hydrazine functional groups.

A. *Schematic of ELP chemical modification steps to achieve bioconjugation of hydrazine groups.* ***B.*** *Representative ^1H NMR (DMSO solvent) spectra of final ELP-hydrazine polymer (purple) and Boc-*

protected intermediate (green). The modification efficiency was determined by comparing the integration of methyl protons of the Boc group (δ = 1.46 and 1.39, 27H) with the aromatic protons of tyrosine (δ = 7.00 and 6.62, 4H) in Boc-protected ELP. Removal of Boc groups was also verified by ^{1}H NMR. The Boc protecting group is completely removed from the hydrazine in ELP-hydrazine. ***C.*** *Calculation of ELP hydrazine modification.*

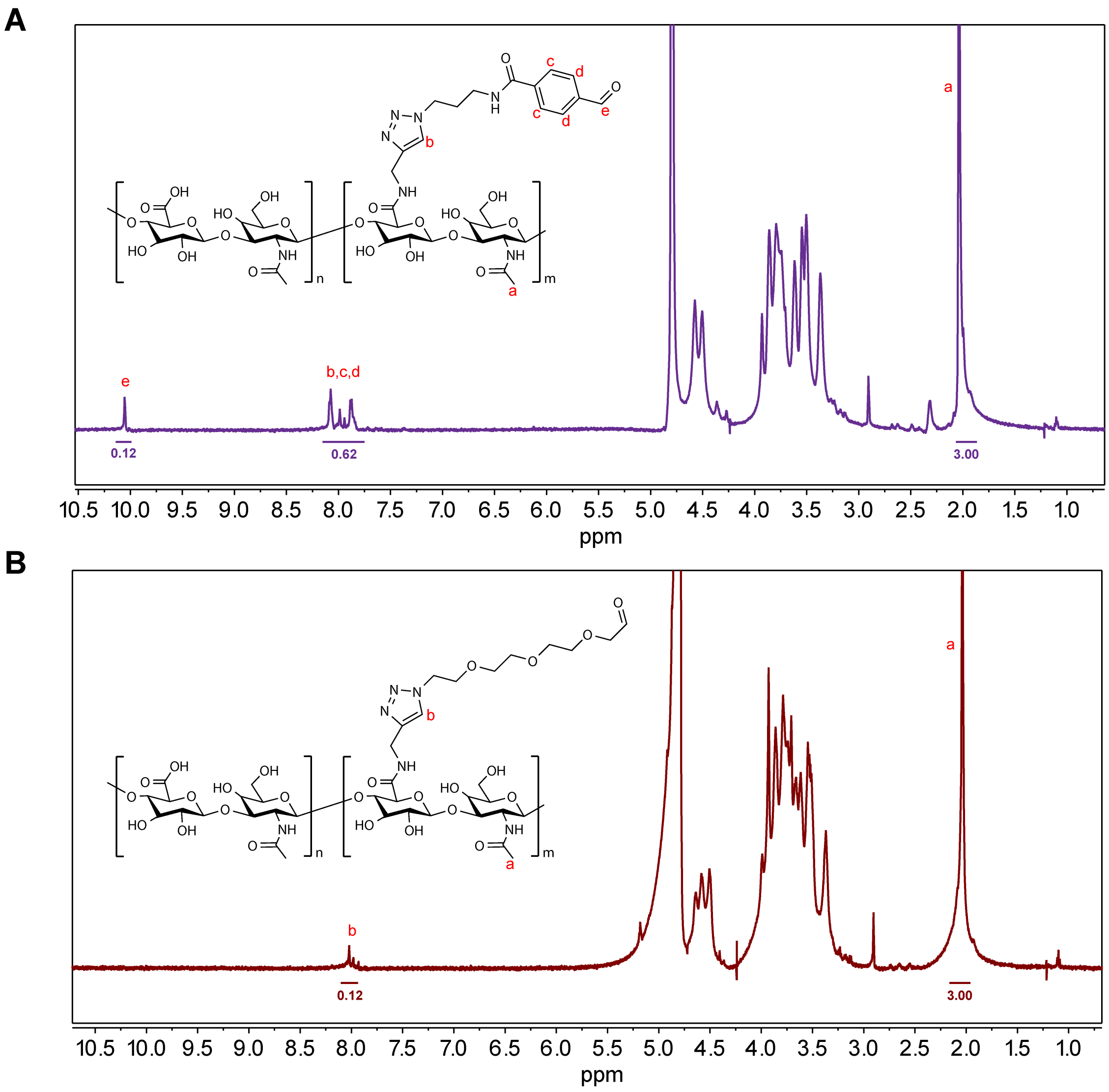

Fig. S3. Hyaluronan (HA) modified with benzaldehyde or aldehyde functional groups.
A. *Representative ^{1}H NMR (D_2O solvent) spectra of 12% modified HA-benzaldehyde polymer. The modification efficiency was determined by measuring the protons on the benzene ring (δ = 7.93 and 7.82, 4H), triazole linkage (δ = 7.9, 1H), and aldehyde group (δ = 9.9, 1H) relative to the protons of the acetyl group (δ = 1.8, 3H).* ***B.*** *Representative ^{1}H NMR (D_2O solvent) spectra of 12% modified HA-aldehyde polymer. The modification efficiency was determined by measuring the protons on the triazole linkage (δ = 7.9, 1H) relative to the protons of the acetyl group (δ = 1.8, 3H).*

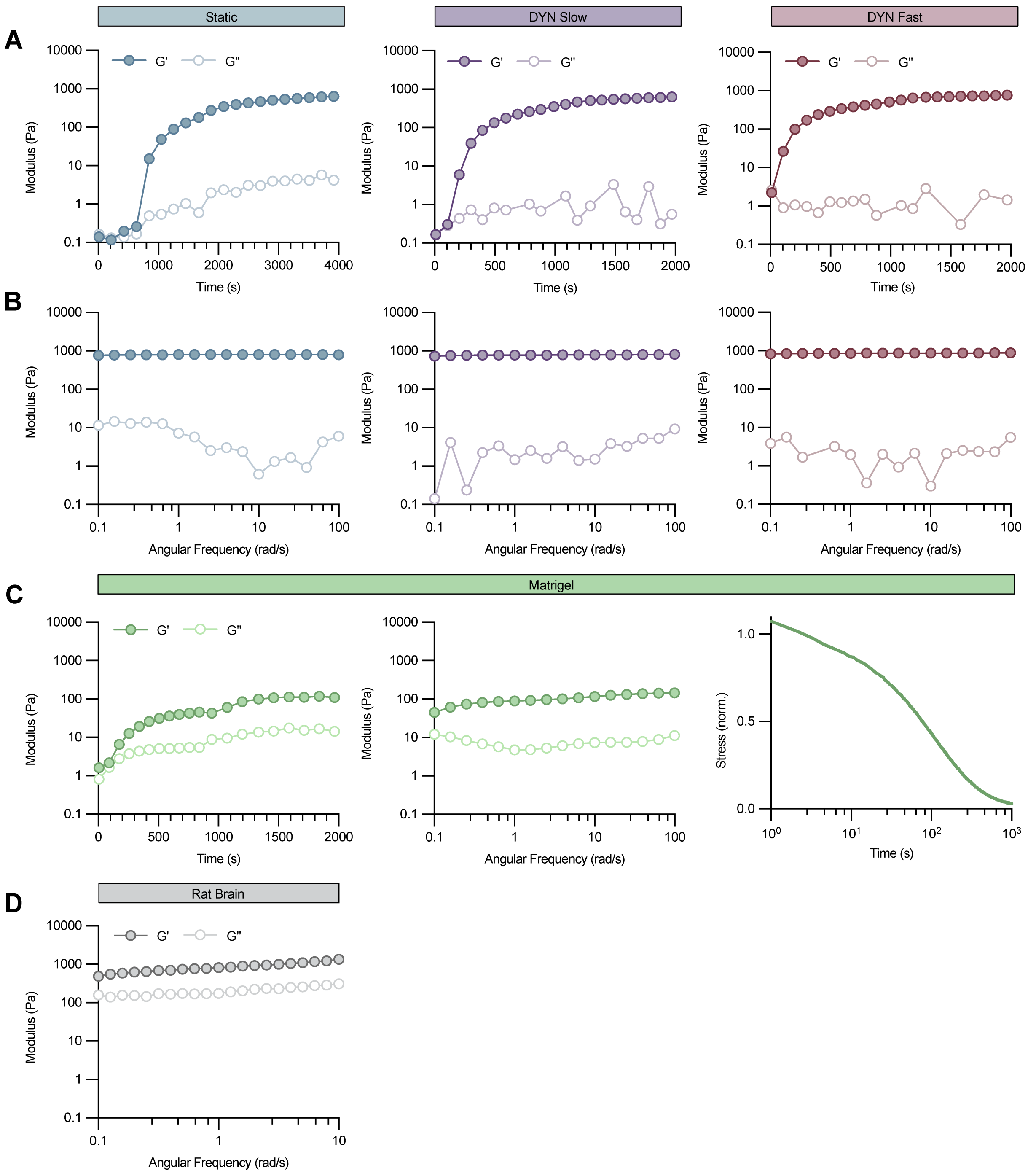

Fig. S4. Characterization of hydrogel rheological properties.

A. *Representative gelation time sweeps of HELP gels (Static, DYN Slow, DYN Fast) showing storage moduli (G', filled symbols) and loss moduli (G", open symbols) during crosslinking.* ***B.*** *Representative frequency sweeps performed at a fixed strain of 1% showing storage moduli (G', filled symbols) and loss moduli (G", open symbols) for all gel formulations.* ***C.*** *Characterization of Matrigel rheological properties. Left: Representative gelation time sweep. Center: Representative frequency sweep at fixed*

strain of 1%. Right: Normalized stress relaxation curve at a fixed strain of 10%. ***D.*** *Representative frequency sweep performed at a fixed strain of 1% for rat brain tissue.*

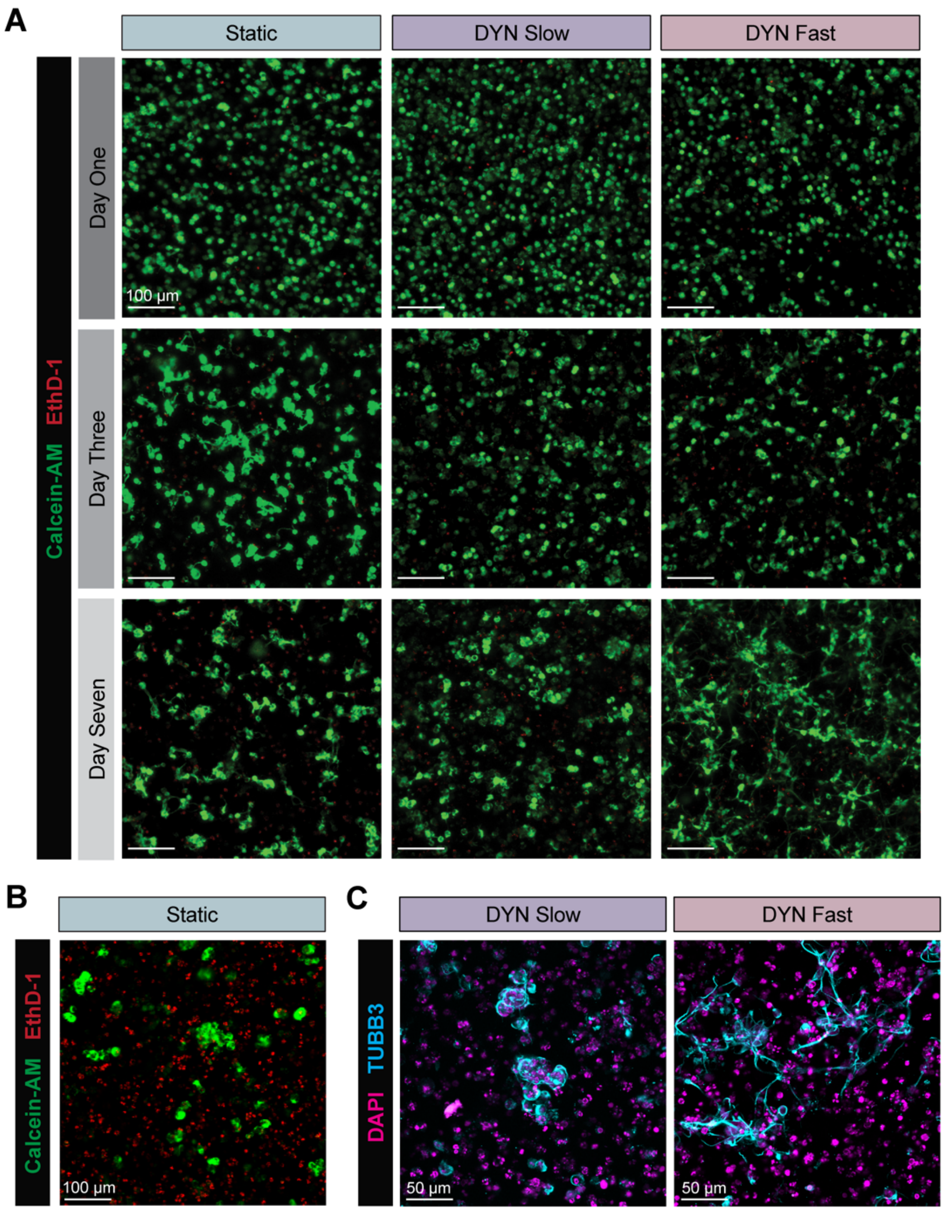

Fig. S5. Human NPC viability in HELP over time.

A. *Representative maximum projection fluorescence images of hiPSC-derived NPCs encapsulated at 3 x 10^4 cells/µL within all three gel formulations after one, three, and seven days of culture with calcein-AM labeled live cells (green) and ethidium homodimer-1 labeled dead cells (red).* ***B.*** *Representative maximum projection fluorescence image of hiPSC-derived NPCs encapsulated at 3 x 10^4 cells/µL within Static HELP gels after 28 days of culture with calcein-AM labeled live cells (green) and ethidium homodimer-1 labeled dead cells (red).* ***C.*** *Representative maximum projection fluorescence images of hiPSC-derived NPC neuritic extension (DAPI-labeled nuclei in magenta, TUBB3-labeled neurites in cyan) after 28 days of culture.*

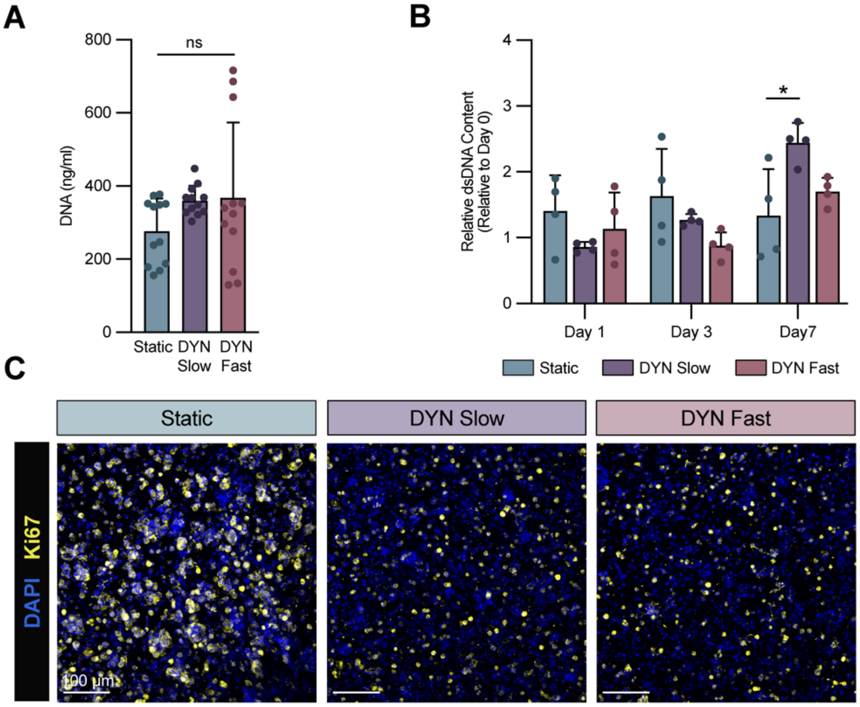

Fig. S6. Human NPCs undergo continuous proliferation in HELP gels.
A. *Quantification of the dsDNA content of encapsulated cells on day zero, one hour post-encapsulation (N = 4 replicate hydrogels, n = 3 technical replicates).* ***B.*** *Quantification of the relative dsDNA content of encapsulated cells on days one, three, and seven (N = 4 replicate hydrogels).* ***C.*** *Representative maximum projection fluorescence images of hiPSC-derived NPCs encapsulated at 3 x 10^4 cells/µL within all three gel formulations after seven days of culture with cells in active phases of the cell cycle (Ki67, yellow).*

*Statistical analyses performed as one-way ANOVA with Tukey's multiple comparisons test. Data plotted as mean ± SD where *p<0.05, **p<0.01, ***p<0.001, ****p<0.0001, and ns = not significant.*

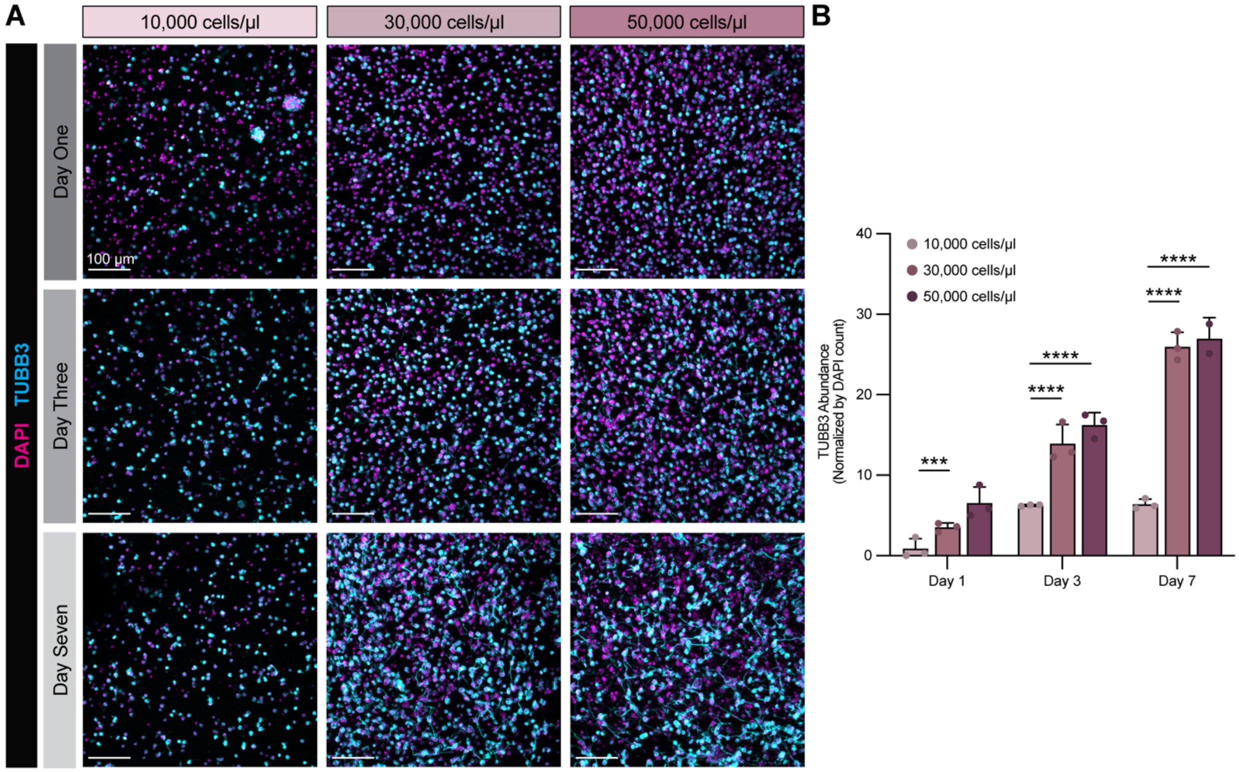

Fig. S7. Neurite extension is dependent on cell density.

A. *Representative maximum projection fluorescence images of hiPSC-derived NPC neuritic extension (DAPI-labeled nuclei in magenta, TUBB3-labeled neurites in cyan) over time at varying cell densities (10k, 30k, and 50k cells/µL).* ***B.*** *Quantification of TUBB3-expressing neurite area, normalized by cell number, over time at varying cell densities (N = 3 replicate hydrogels).*

*Statistical analyses performed as two-way ANOVA with Tukey's multiple comparisons test. Data plotted as mean ± SD where *p<0.05, **p<0.01, ***p<0.001, ****p<0.0001, and ns = not significant.*

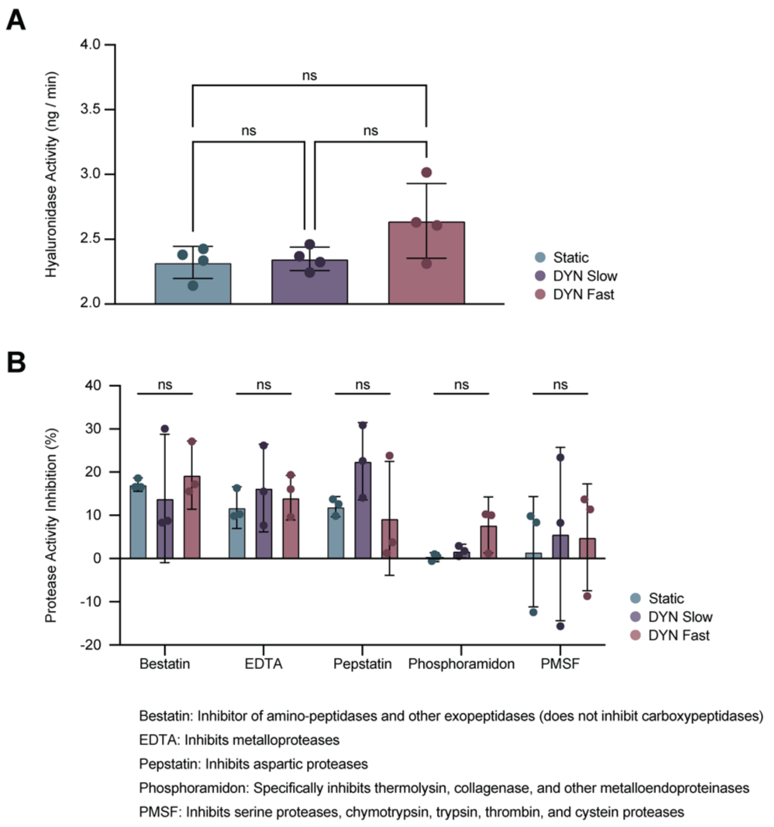

Fig. S8. Human NPC secreted enzyme activity does not vary across gel formulations.
A. *Hyaluronidase activity, measured in ng/mL, of cells within all three gel formulations at day 7 post-encapsulation (N = 4 replicate hydrogels).* ***B.*** *Protease activity at day 7 post-encapsulation was quantified using a commercially available kit (ProteSEEKER), where protease inhibitors and a protease substrate are added to media samples, and the degree of inhibition is dependent on the identity and activity of proteases present in the sample (N = 3 replicate hydrogels).*

*Statistical analyses performed as two-way ANOVA with Šídák's multiple comparisons test. Data plotted as mean ± SD where *p<0.05, **p<0.01, ***p<0.001, ****p<0.0001, and ns = not significant.*

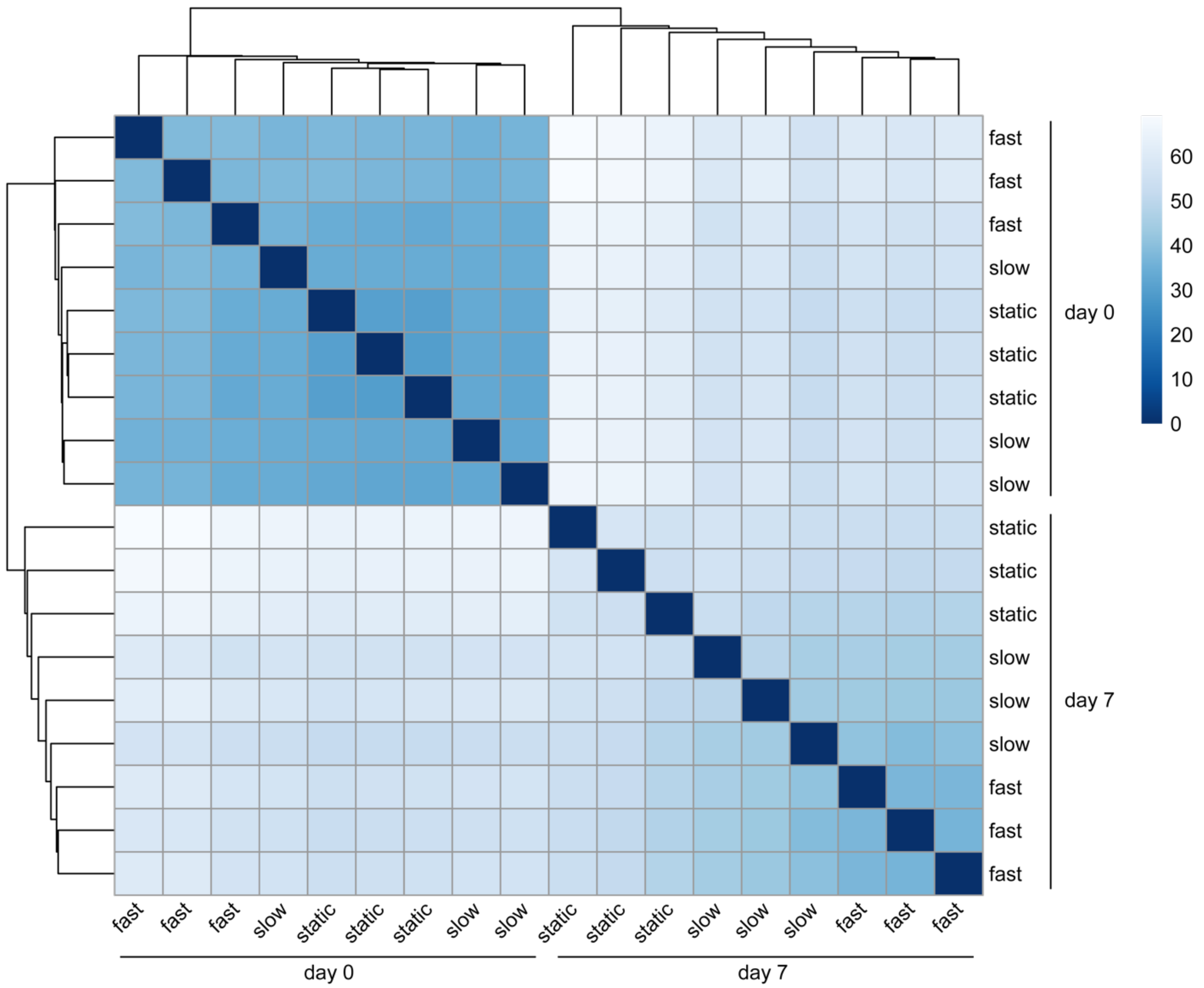

Fig. S9. Sample-to-sample distance map reveals similarities between replicates.
Heatmap of sample-to-sample distances calculated from the variance-stabilizing transformation of count data for overall gene expression.

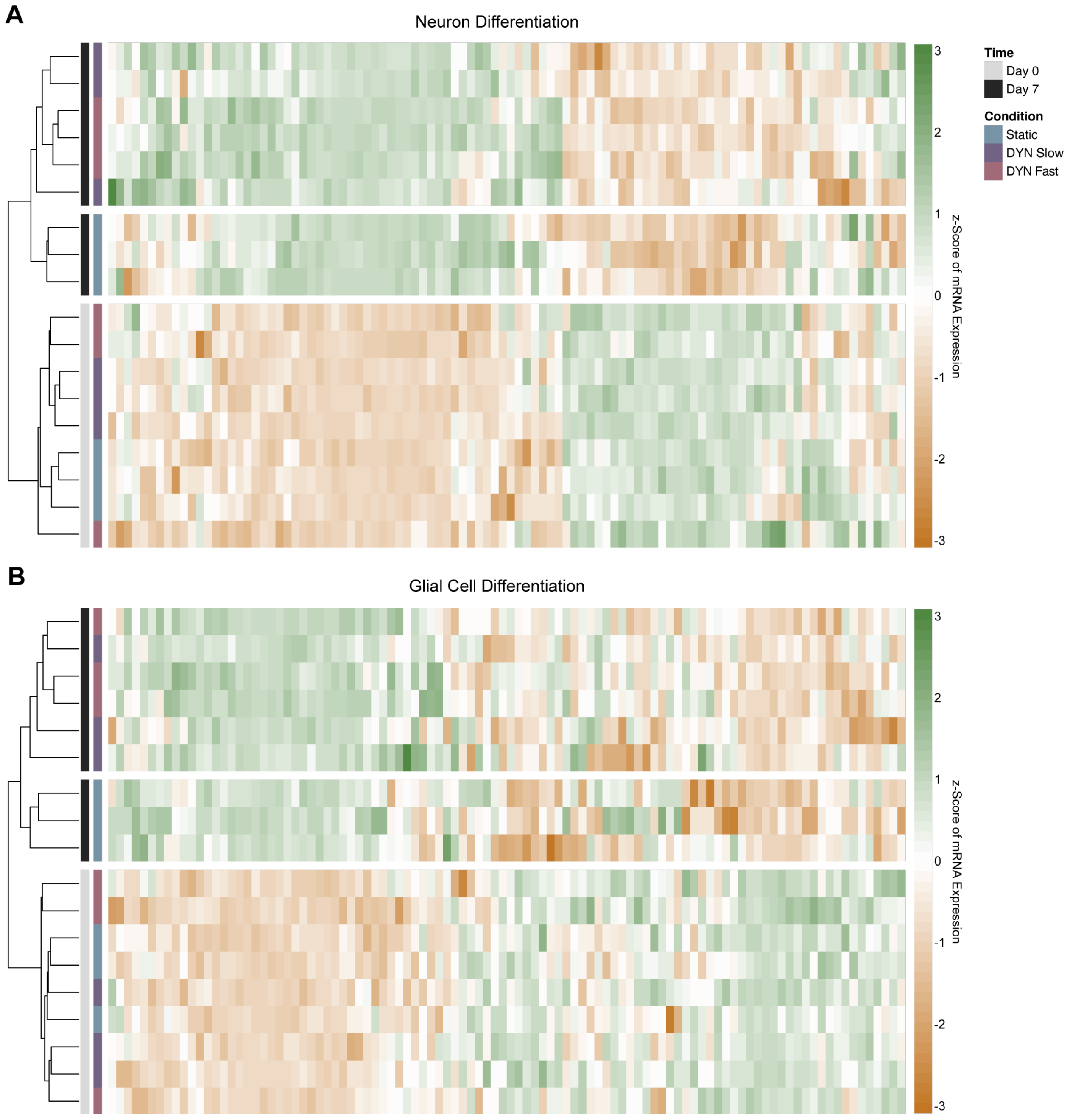

Fig. S10. Transcriptional differences in neuron and glial differentiation genes emerge in hNPCs across time and hydrogel relaxation rate.

A-B. *Heatmap of gene expression represented as Z-scores for the top 100 differentially expressed genes associated with the Gene Ontology term "Neuron Differentiation" (A) and "Glial Cell Differentiation" (B) as identified with DESeq2. Counts were normalized and scaled across columns.*

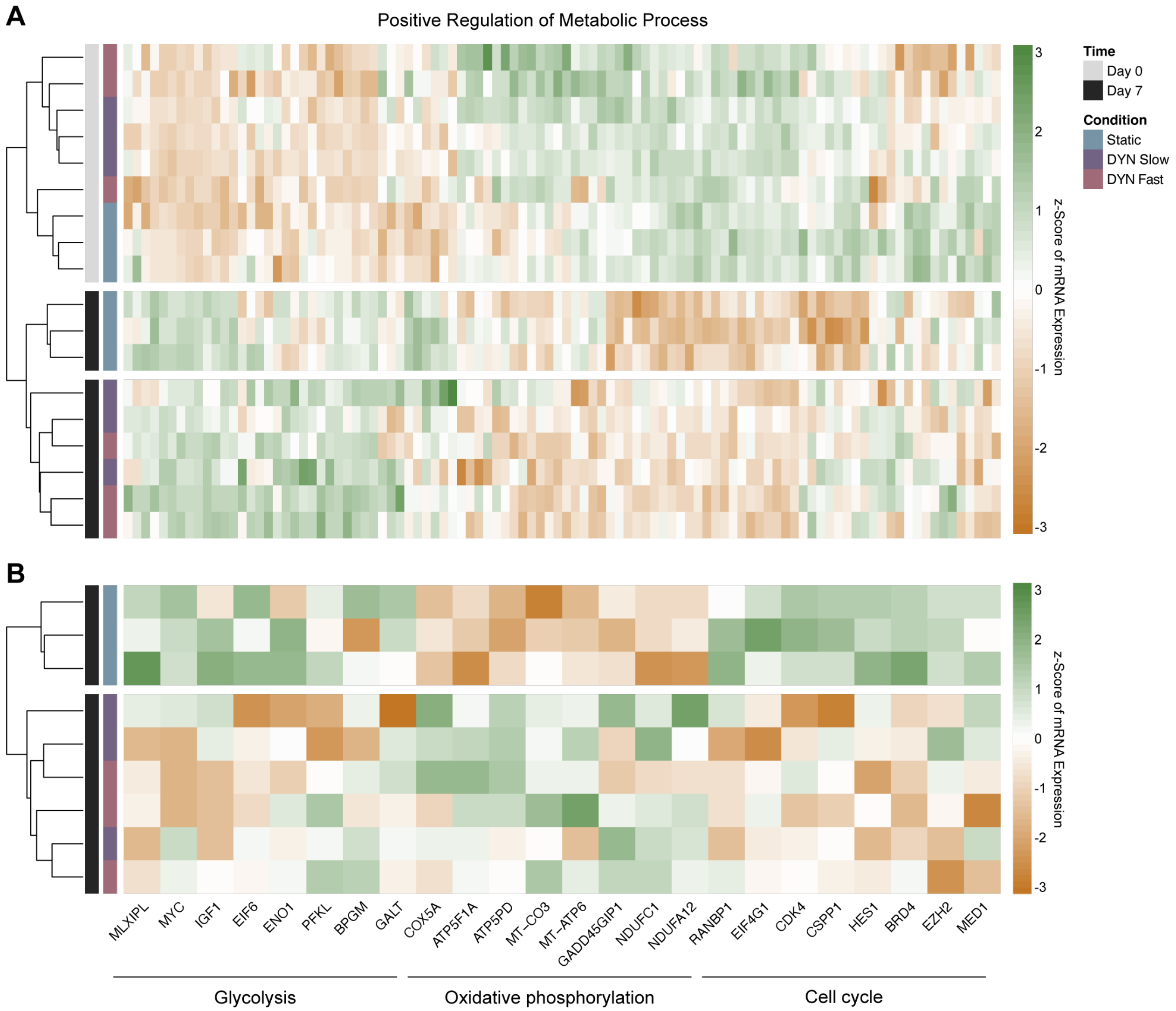

Fig. S11. Transcriptional differences in metabolism-related genes emerge in hNPCs across time and hydrogel relaxation rate.

A. *Heatmap of gene expression represented as Z-scores for the top 100 differentially expressed genes associated with the Gene Ontology term "positive regulation of metabolic process" as identified with DESeq2. Counts were normalized and scaled across columns.* ***B.*** *Heatmap of gene expression represented as Z-scores for genes associated with the Gene Ontology terms "glycolysis", "oxidative phosphorylation", and "positive regulation of cell cycle". Counts were normalized and scaled across columns.*

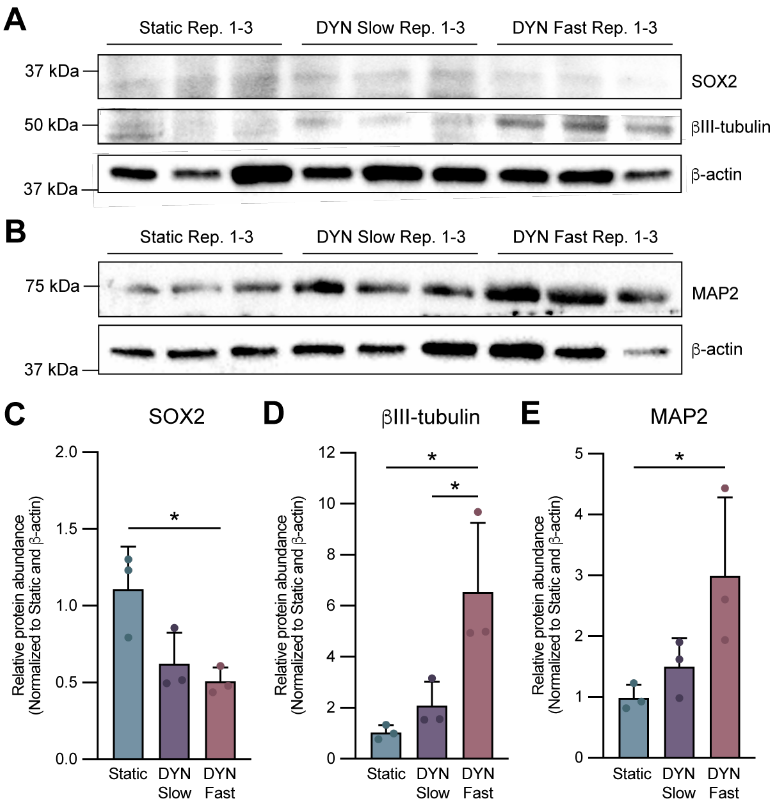

Fig. S12. Viscoelastic hydrogels modulate the protein-level expression of neural lineage markers.

A-B. *Western blot analysis of canonical neural differentiation markers of hiPSC-derived NPCs encapsulated at 3 x 10^4 cells/µL within all three gel formulations after seven days of culture (N = 3 replicate hydrogels). β-actin expression was used as a loading control.* ***C-E.*** *Western blot quantification of data in* ***A-B****.*

*Statistical analyses performed as one-way ANOVA with Tukey's multiple comparisons test. Data plotted as mean ± SD where *p<0.05, **p<0.01, ***p<0.001, ****p<0.0001, and ns = not significant.*

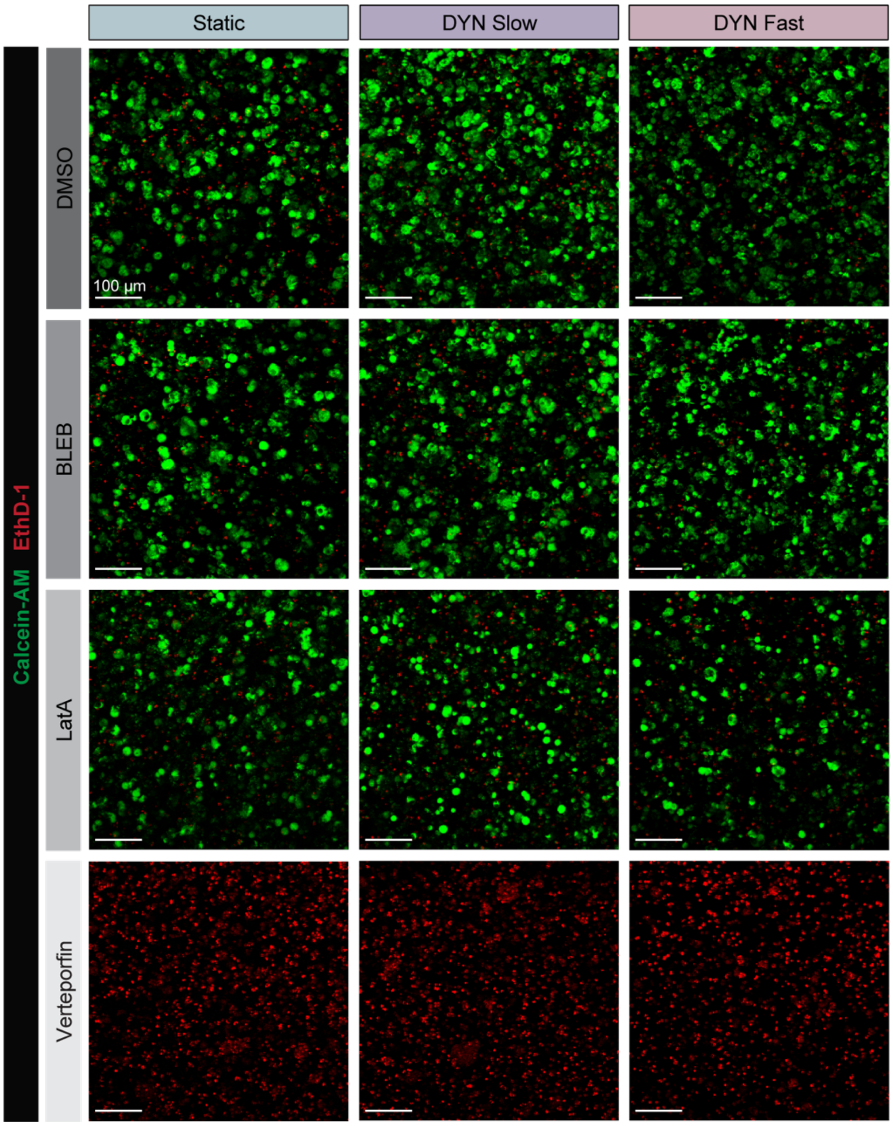

Fig. S13. Viability of hNPCs in response to chemical inhibitors of the cytoskeleton.
Representative maximum projection fluorescence images of hiPSC-derived NPCs encapsulated within all three gel formulations after seven days of culture and following treatment with blebbistatin (BLEB), latrunculin A (LatA), and Verteporfin (4 µM, negative control) *with calcein-AM labeled live cells (green) and ethidium homodimer-1 labeled dead cells (red).*

Table S1. List of primer sequences.

Target	Forward Primer (5' to 3')	Reverse Primer (5' to 3')
**GAPDH**	CATGAGAAGTATGACAACAGCCT	AGTCCTTCCACGATACCAAAGT
**SOX2**	TTCACATGTCCCAGCACTACCAGA	TCACATGTGTGAGAGGGGCAGTGTGC
**NESTIN**	CAGCGTTGGAACAGAGGTTGG	TGGCACAGGTGTCTCAAGGGTAG
**PAX6**	TGCTGCACATTTGGAGACAC	TGCAGAAATGTGCTGCTGTG
**EOMES**	GCCATGCTTAGTGACACCGA	GGACTGGAGGTAGTACCGC
**TBR1**	GCAGCAGCTACCCACATTCA	AGGTTGTCAGTGGTCGAGATA
**TUBB3**	GGCCAAGGGTCACTACACG	GCAGTCGCAGTTTTCACACTC
**MAP2**	CCACCTGAGATTAAGGATCA	GGCTTACTTTGCTTCTCTGA
**RBFOX3**	CCAAGCGGCTACACGTCTC	CGTCCCATTCAGCTTCTCCC

Table S2. Statistical details (separate file).
Full statistical details for each figure panel.
